# Quinolinonyl Non-Diketo Acid Derivatives as Inhibitors of HIV-1 Ribonuclease H and Polymerase Functions of Reverse Transcriptase

**DOI:** 10.1021/acs.jmedchem.1c00535

**Published:** 2021-06-09

**Authors:** Antonella Messore, Angela Corona, Valentina Noemi Madia, Francesco Saccoliti, Valeria Tudino, Alessandro De Leo, Davide Ialongo, Luigi Scipione, Daniela De Vita, Giorgio Amendola, Ettore Novellino, Sandro Cosconati, Mathieu Métifiot, Marie-Line Andreola, Francesca Esposito, Nicole Grandi, Enzo Tramontano, Roberta Costi, Roberto Di Santo

**Affiliations:** † Dipartimento di Chimica e Tecnologie del Farmaco, Istituto Pasteur-Fondazione Cenci Bolognetti, “Sapienza” Università di Roma, p.le Aldo Moro 5, I-00185 Rome, Italy; ‡ Department of Life and Environmental Sciences, University of Cagliari, Cittadella Universitaria di Monserrato, SS554-09042 Monserrato (CA), Italy; § D3 PharmaChemistry, Italian Institute of Technology, Via Morego 30, I-16163 Genova, Italy; ∥ Department of Environmental Biology, “Sapienza” University of Rome, p.le Aldo Moro 5, I-00185 Rome, Italy; ⊥ DiSTABiF, University of Campania “Luigi Vanvitelli”, Via Vivaldi 43, 81100 Caserta, Italy; # Department of Pharmacy, University Federico II of Naples, Via D. Montesano 49, 80131 Naples, Italy; ∇ Laboratoire MFP, UMR 5234, CNRS - Université de Bordeaux, 146 rue Léo Saignat, 33076 Bordeaux cedex, France

## Abstract

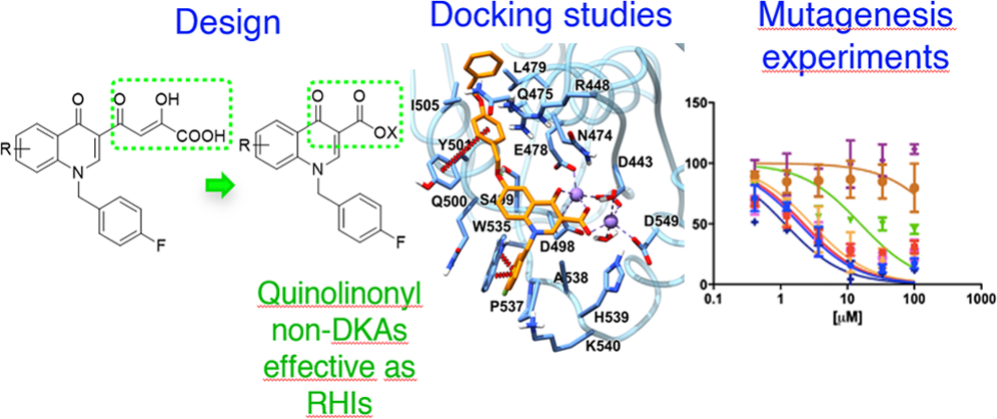

Novel anti-HIV agents are still needed to overcome resistance issues, in particular inhibitors acting against novel viral targets. The ribonuclease H (RNase H) function of the reverse transcriptase (RT) represents a validated and promising target, and no inhibitor has reached the clinical pipeline yet. Here, we present rationally designed non-diketo acid selective RNase H inhibitors (RHIs) based on the quinolinone scaffold starting from former dual integrase (IN)/RNase H quinolinonyl diketo acids. Several derivatives were synthesized and tested against RNase H and viral replication and found active at micromolar concentrations. Docking studies within the RNase H catalytic site, coupled with site-directed mutagenesis, and Mg^2+^ titration experiments demonstrated that our compounds coordinate the Mg^2+^ cofactor and interact with amino acids of the RNase H domain that are highly conserved among naïve and treatment-experienced patients. In general, the new inhibitors influenced also the polymerase activity of RT but were selective against RNase H vs the IN enzyme.

## Introduction

The human immunodeficiency virus type 1 (HIV-1) is the agent responsible for the acquired immunodeficiency syndrome (AIDS). According to the last estimates by the World Health Organization (WHO) and the Joint United Nations Programme on HIV and AIDS (UNAIDS), globally, there were 38 million people living with HIV in 2018 and only 62% of them were receiving antiretroviral treatment by the end of 2018.^1^

In total, 44 Food and Drug Administration (FDA)-approved medicines can be used in the treatment of HIV, including multiclass combination products, nucleoside reverse transcriptase (RT) inhibitors (NRTIs), non-nucleoside RT inhibitors (NNRTIs), protease inhibitors (PIs), integrase (IN) inhibitors (INSTIs), fusion inhibitors, CCR5 antagonists, postattachment inhibitors, and pharmacokinetic enhancers.^2^ Treatment with HIV medicines is called antiretroviral therapy (ART), which involves taking a combination of drugs as a single pill or in various pill combinations and which generally comprehends combinations of at least three drugs from different HIV drug classes (usually NRTIs, NNRTIs, and INSTIs).^2,3^ These approaches have resulted in suppression of viral replication, with decreased death rates^4^ and morbidity.^5^ Still, therapy suspension or lack of adherence is associated with a rapid viral rebound because such therapies do not affect the viral reservoir of latently infected cells, being the main obstacle to viral eradication.

Despite the undisputed advantage of ART, this therapy still has several drawbacks, which include long-term toxicity and drug–drug interactions.^6^ Moreover, life-long treatment strongly impairs the adherence, drastically promoting the selection of variants of the virus resistant to current therapies.^7^ This resistance phenomenon represents the major clinical challenge in the fight against AIDS. Therefore, new anti-HIV agents are still urgently needed, in particular, inhibitors acting against novel viral targets that can contribute overcoming the resistance issue.^8−10^

Since the discovery of HIV, RT has been the first exploited therapeutic target. RT is an RNA-dependent DNA polymerase that utilizes a strand of RNA to synthesize double-stranded viral DNA that can eventually integrate into the genome of the infected cell.^11^ It is a multifunctional enzyme with DNA polymerase RNA- and DNA-dependent (RDDP and DDDP, respectively) and endonuclease (ribonuclease H, RNase H) activities. RNase H function is essential for virus replication since it specifically cleaves the RNA moiety of the RNA/DNA hybrid to generate a DNA duplex to be integrated into the host cell. The RNase H active site contains a highly conserved DEDD motif consisting of four carboxylate amino acid residues in close proximity (D443, E478, D498, and D549) that interact with two Mg^2+^ ions.^11^ It is worthy of note that a similar arrangement is observed in the active site of HIV-1 IN, another metalloenzyme that plays critical roles in viral infection. Indeed, three highly conserved residues in the catalytic core domain of this enzyme (D64, D116, and E152; DDE motif) coordinate the two Mg^2+^ ions necessary for its trans-esterase activity.^12^

Despite being a valid and promising drug target, RNase H inhibitors have not reached the clinical pipeline yet. Indeed, all of the RT-targeting drugs approved thus far are inhibitors of the RDDP activity and the development of RNase H inhibitors (RHIs) has lagged behind so that no drug targeting RNase H has been approved yet. This can be attributed to two reasons: (i) the availability of expertise on inhibitors of other DNA polymerases^13^ that encouraged the development of drugs targeting the RT-associated RDDP function, and (ii) the open morphology of the RNase H function that is hard to target and showing a strong competition with the substrate for access to the catalytic core.^14^

However, RNase H plays a key role in the viral life cycle and shows a high degree of conservation of the entire domain upon naïve and treatment-experienced patients.^15^ Thus, more recently, efforts were boosted in the development of new RHIs as relevant to enhance the antiretroviral armory and potentially able to counteract circulating HIV-1 strains resistant to the approved drugs.^15−17^ In recent years, the development of more effective screening techniques^18,19^ and the availability of more and more detailed structural data helped design and identify new inhibitors that can be grouped into two main categories: active-site and allosteric inhibitors. The first ones are small molecules that showed RNase H inhibitory activity at low micromolar or submicromolar ranges. These inhibitors mainly contain a hydrophobic moiety linked to a two-metal-cation chelating core, an element reminiscent of that observed for canonical HIV-1 INSTIs.^20−23^ This moiety plays a key role as a driving force for the binding, conferring a high potency of inhibition, but leads to limited selectivity towards RT-associated RDDP and/or IN activities.^20−22,24^

In this field of research, our group has previously reported DKA derivatives that proved to be dual inhibitors of IN and RH^25−27^ or INSTIs endowed with marginal RH inhibition activities.^10,28^

**Figure 1:**
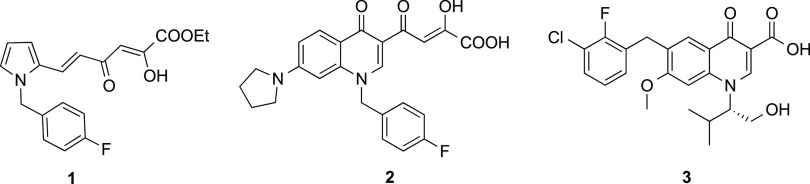
Inhibitors of HIV-1 RNase H function of RT and/or IN enzymes.

Among the dual inhibitors, an example is the pyrrolyl diketo hexenoic ester (RDS 1643, **1**), which was the first DKA derivative reported as RHI to have an antiviral effect^29−32^ (Figure 1), further developed on RDS 1759,^25^ with a selective mode-of-action and the ability to target conserved residues within the RT RNase H domain. Conversely, quinolinonyl diketo butanoic derivative **2** (Figure 1) showed a more prominent IN inhibitory activity with respect to that against RNase H, reporting IC_50_ values of 0.028 and 5.1 μM, respectively.^33^

Despite the relevance of the DKA branch in the inhibitory activity, it is well-known that the DKA chain suffers from several limits related to the pharmacokinetic and pharmacodynamic profiles. Indeed, the DKA chain is responsible for the poor solubility, high metabolic turnover, and low permeability through the cell membrane of molecules containing such a chain. Furthermore, a time-dependent decrease in the activity in solution at room temperature has been proven, even during short periods.^30−34^ Therefore, to overcome the limits of the DKA moiety, a variety of compounds were developed by transferring the DKA chain to scaffolds characterized by improved druglike qualities that dialed out the undesirable DKA properties. A notable example is the INSTI elvitegravir (**3**, Figure 1) approved by the FDA as a successful anti-HIV drug. In this compound, the DKA chain was shortened into a carboxylic acid function that, together with the ketone group in the 4-position of the quinolinone ring, chelates the two Mg^2+^ ions within the IN catalytic site.^35^

**Figure 2:**
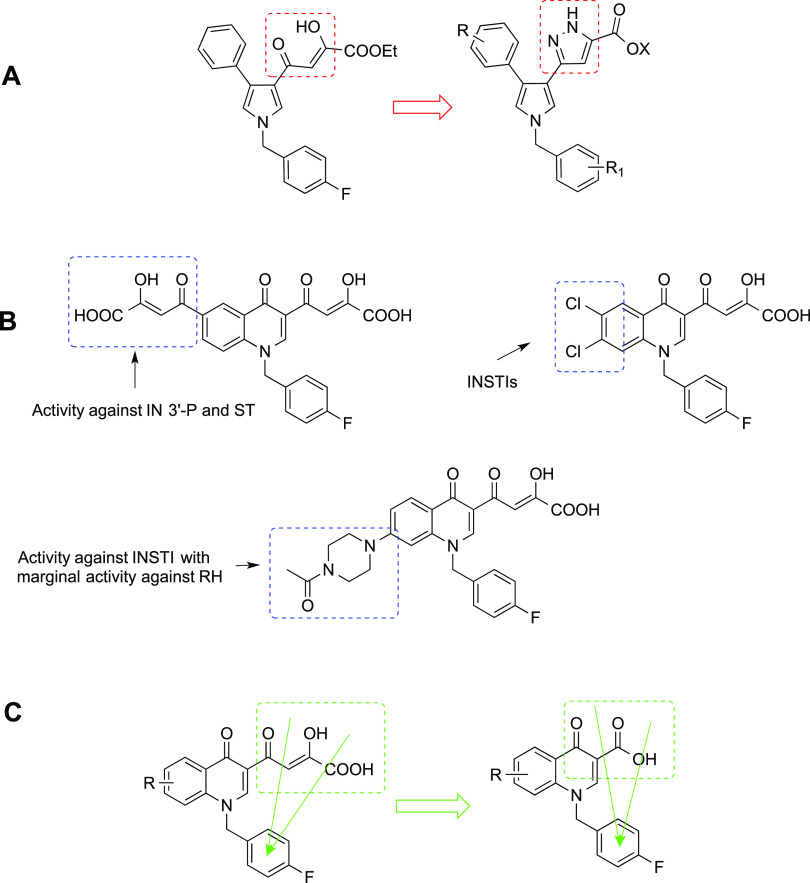
Design of pyrrolyl pyrazole carboxylates as RH inhibitors (**A**), quinolinones as INIs (**B**), and the new quinolinonyl non-DKA derivatives as RHIs (**C**).

Recently, we successfully obtained a new selective RHIs by design of pyrrolyl pyrazole carboxylic acids.^36^ This new scaffold has been achieved converting the diketo group of our previously reported dual IN/RH inhibitors pyrrolyl DKA derivatives^25−27^ into a pyrazole moiety (Figure 2A). In this way, we obtained compounds active at micromolar/submicromolar concentrations against RNase H and selective for RNase H vs IN from 5 to >18 times. Also, these non-DKA inhibitors blocked the viral replication and proved to interact with conserved residues within the RNase H active site domain.^36^

Besides pyrrolyl DKA inhibitors, we also reported quinolinonyl DKA derivatives. We recently designed a few series of quinolinones and defined the structural elements that were vital in influencing the activity. Indeed, the bifunctional DKA derivatives were IN inhibitors nonselective against 3′-processing *vs* strand transfer steps,^37^ while the quinolinones with small substituents on 6- and 7-positions gave potent INSTIs,^28^ and finally, the basic quinolinones bearing an amino substituent in the 7-position resulted in IN inhibitors with marginal activity against RH (nM against ST and >10 μM against RH) (Figure 2B).^10^

In this paper, we applied an isosteric approach to those quinolonyl DKAs, with the aim to obtain a new class of compounds endowed with selective inhibitory activity against RNase H. We referred to integrase inhibitor **3**, conceived as a DKA isoster capable of chelating ions within the catalytic site. Thus, starting from quinolonyl DKA, we designed non-DKA quinolinonyl derivatives in which the DKA unit is replaced by the carboxylic unit in the 3-position and the carbonyl group in the 4-position of the quinolone moiety. In this way, we retained the chelating unit capable of binding the ions within the catalytic site. At the same time, we changed the distance between the benzyl moiety and the chelating group, critical to achieving optimal interaction with the IN catalytic site, with the aim to produce selectivity toward the RH function of the RT (Figure 2C).

**Chart 1:**
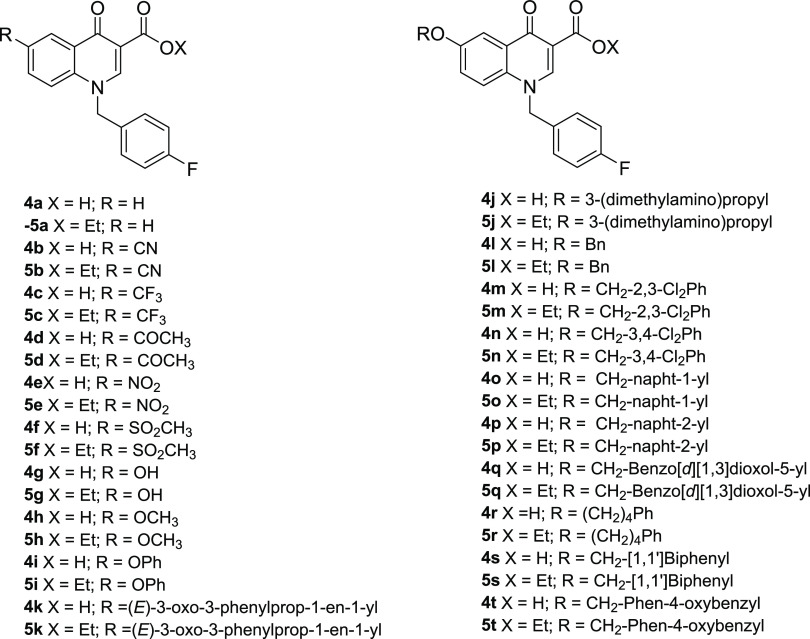
Structures of the Newly Designed Quinolinonyl Derivatives **4a–t** and **5a–t**

All of the newly designed compounds **4a–t** and **5a–t** are characterized by the introduction of different substituents in the 6-position of the quinolinone ring (Chart 1). In detail, we introduced (i) a hydrogen atom or a hydroxyl group; (ii) various ether groups characterized by different degrees of freedom or steric hindrance as methoxy, phenoxy, aminoalkyloxy, and arylmethyloxy; (iii) acetyl, cyano, nitro, trifluoromethyl, and methylsulfonyl groups; and (iv) a phenylpropenone moiety.

The newly synthesized compounds have been evaluated *in vitro* for their ability to inhibit the specific enzymatic activity of recombinant RNase H, for their cytotoxicity and antiviral activity against HIV-1 in human cells. Besides, a rationalization of the interaction with the biological target has been proposed, based on docking studies using the crystal structure of RNase H, and validated by site-directed mutagenesis on the residues indicated as being crucial for the binding. Finally, selected derivatives have been tested for their activity against IN and RDDP functions of the RT to evaluate their selectivity.

## Results and Discussion

### Chemistry

**Scheme 1:**
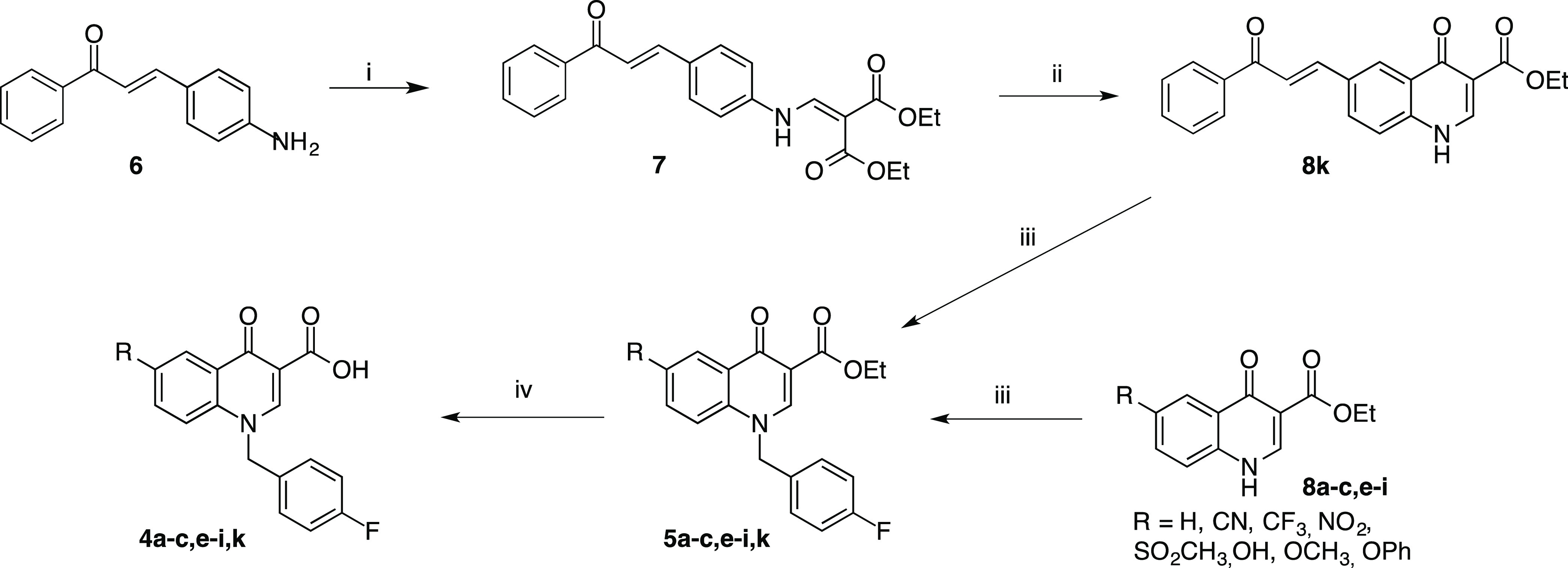
Synthetic Route to **4a–c,e–i,k** and **5a–c,e–i,k** Derivatives Reagents and conditions: (i) EMME, 90 °C, 3 h, 90% yield; (ii) diphenyl ether, reflux, 2 h, 100% yield; (iii) 4-fluorobenzyl bromide, K_2_CO_3_, *N*,*N*-dimethylformamide (DMF), 100 °C, 2–3 h, 70–93% yield; (iv) proper base, 1:1 tetrahydrofuran (THF)/EtOH, reflux or room temp, 1–4 h, 80–100% yield.

Compounds **4d** and **5d** were obtained as already reported.^38^ The synthesis of derivatives **4a–c,e–i,k** and **5a–c,e–i,k** is outlined in Scheme 1. Condensation of (*E*)-3-(4-aminophenyl)-1-phenylprop-2-en-1-one^39^ (**6**) with diethyl ethoxymethylenemalonate (EMME) gave intermediate **7**, which was submitted to thermal ring closure to give **8k** (Gould–Jacobs reaction).^40^ Derivatives **5a–c,e–i,k** were obtained by alkylation in position 1 of the proper quinolinonyl derivative **8a–c,e–i**(41−49) or **8k** with *p*-fluorobenzyl bromide in the alkaline medium. The subsequent base-catalyzed hydrolysis of ester derivatives **5a–c,e–i,k** afforded the corresponding acids **4a–c,e–i,k**.

**Scheme 2:**
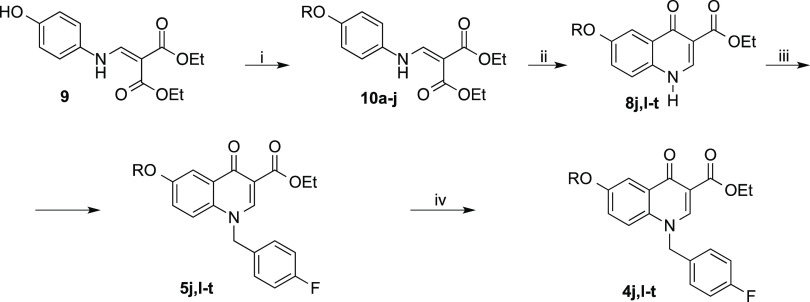
Synthetic Route to **4j,l–t** and **5j,l–t** Derivatives Reagents and conditions: (i) appropriate halide, *t*-BuOK, DMF, 0 °C to room temp, 2–3 h, 60–90% yield; (ii) diphenyl ether, reflux, 2–3 h, 90–100% yield; (iii) 4-fluorobenzyl bromide, K__2__CO__3__, DMF, 100 °C, 2–3 h, 40–90% yield; (iv) NaOH 20%, 1:1 THF/EtOH, reflux, 1–2 h, 50–100% yield.

The synthesis of compounds **4j,l–t** and **5j,l–t** was performed as reported in Scheme 2. The synthetic approach resembles the one described above for compounds **4a–c,e–i,k** and **5a–c,e–i,k**. Noteworthy, the synthetic pathway starts with an *O*-alkylation of diethyl 2-(((4-hydroxyphenyl)amino)methylene)malonate^50^ (**9**) with the appropriate alkyl halide to obtain intermediates **10a–j**.

### Evaluation of Biological Activities

#### *In Vitro* Screening for RNase H Inhibitory Activity

All compounds **4a–t** and **5a–t** were tested *in vitro* in enzyme inhibition assays against recombinant RNase H (Table 1), using the known RNase H inhibitors RDS 1759^25^ (**11**) and β-thujaplicinol (BTP) used as positive controls. The assays were performed in conditions of competition with the substrate, without preincubation, starting the reaction by adding the enzyme, to avoid overestimation of compound potency and proving that it was not affected by substrate displacement, as reported for other active compounds.^14,51^

**Table 1:**
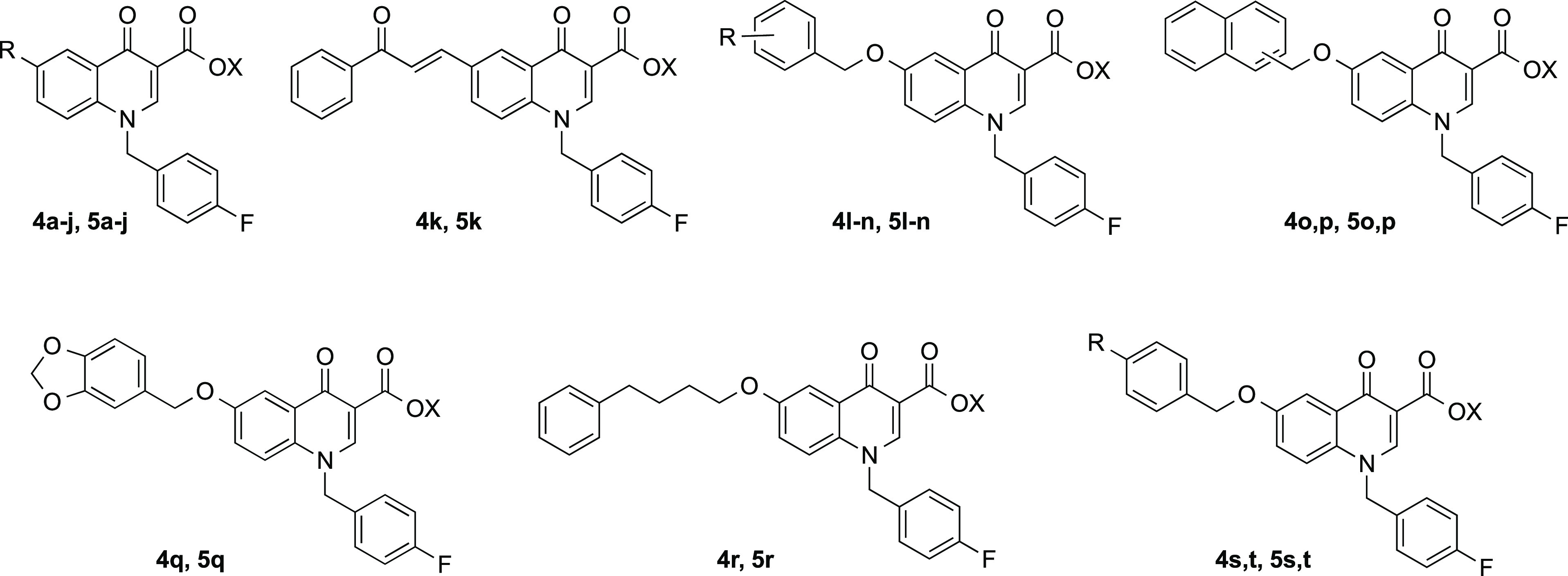
Enzymatic Activities on RNase H of the Newly Synthesized Compounds **4a–t** and **5a–t**

cpd	X	R	anti-RH activity (IC_50_ ± SD)^a^
**4a**	H	H	>100
**4b**	H	CN	>100
**4c**	H	CF_3_	>100
**4d**	H	COCH_3_	16.3 ± 1.42
**4e**	H	NO_2_	56.0 ± 4
**4f**	H	SO_2_CH_3_	100
**4g**	H	OH	74 ± 9
**4h**	H	OCH_3_	>100
**4i**	H	OPh	32.0 ± 11.0
**4j**	H	O(CH_2_)_3_N(CH_3_) _2_	15.4 ± 3.0
**4k**	H		5.9 ± 0.6
**4l**	H	H	34.0 ± 0.1
**4m**	H	2,3-Cl_2_	8.19 ± 0.05
**4n**	H	3,4-Cl_2_	29.5 ± 0.5
**4o**	H	1-yl	1.51 ± 0.21
**4p**	H	2-yl	30.3 ± 1.7
**4q**	H		13.5 ± 1.7
**4r**	H		8.27 ± 0.45
**4s**	H	Ph	7.47 ± 1.55
**4t**	H	OBn^b^	7.48 ± 0.28
**5a**	Et	H	nt^c^
**5b**	Et	CN	>100
**5c**	Et	CF_3_	59.0 ± 5.0
**5d**	Et	COCH_3_	11.0 ± 1.0
**5e**	Et	NO_2_	47.8 ± 10
**5f**	Et	SO_2_CH_3_	>100
**5g**	Et	OH	>100
**5h**	Et	OCH_3_	>100
**5i**	Et	OPh	45.5 ± 1.5
**5j**	Et	O(CH_2_)_3_N(CH_3_)_2_	>100
**5k**	Et		15.3 ± 1.6
**5l**	Et	H	8.0 ± 1.6
**5m**	Et	2,3-Cl_2_	24.0 ± 4.9
**5n**	Et	3,4-Cl_2_	37.2 ± 7.6
**5o**	Et	1-yl	1.49 ± 0.33
**5p**	Et	2-yl	19.6 ± 0.05
**5q**	Et		55.3 ± 1.7
**5r**	Et		28.4 ± 4.3
**5s**	Et	Ph	>100
**5t**	Et	OBn^b^	>100
**11**(25)			7.50 ± 1.32
**BTP**^d^			0.19 ± 0.03

a Inhibitory concentration 50% (μM) determined from dose-response curves: experiments performed against HIV-1 RT-associated RNase H activity.

b Bn, benzyl.

c nt, not tested.

d BTP, β-thujaplicinol.

In general, the newly designed quinolinones were proven active against RH, with 27 out of 39 tested compounds showing measurable IC_50_ under 100 μM concentration. Moreover, 20 compounds were active at concentrations up to 34 μM, and 8 compounds were active in the low micromolar range. The most active compounds of the series were **4o** and **5o** having comparable IC_50_ values (about 1.5 μM).

The acid derivatives were generally more active than the ester counterparts, although notable exceptions can be cited like the equipotent couples **4d–5d**, **4o–5o**, and **4e–5e** and the case of ester **5l** being more active than the acid counterpart **4l**.

In general, compounds with small substituents (**4a–h** and **5a–h**) were found to be active in the high micromolar range (IC_50_ ≥ 50 μM) or were inactive, with the sole exception of the couple **4d** and **5d** showing IC_50_ values 16.3 and 11.0 μM, respectively.

Indeed, within this subseries, the best acting compound, **5e**, showed a decrease of 3–4-fold in activity with respect to the acetyl counterparts **4d** and **5d**.

The removal of the substituent in position 6 of the quinolinonyl ring led to a loss of activity, as observed for the acid **4a**. Similarly, by replacing the acetyl group proper of compounds **4d** and **5d** with a hydroxyl one, a decrease in inhibitory potency was observed (**5g,** IC_50_ > 100 μM; **4g**, IC_50_ = 74 μM). It is also worthy of note that the methylation of compounds **4g** and **5g** led to derivatives **4h** and **5h**, which reported no activity.

An increase in the dimension of the substituent gave compounds endowed with better activity. Indeed, within the ether subseries **4h–j** and **5h–j,** the methoxy compounds **4h** and **5h** were inactive, phenyl ethers **4i** and **5i** reported a moderate inhibition with IC_50_ values of 32.0 and 45.5 μM, respectively, while the dimethylaminopropyl derivative **4j** showed good efficacy (IC_50_ = 15.4 μM). Definitively, among the acid **4h–j**, it is possible to notice that the activity increases in the following order: **4h** < **4i** < **4j**.

A further increase in the moiety placed in the 6-position of the quinolinone ring, like the one with the phenylpropenone substituent (**4k** and **5k)**, gave good inhibitory potencies. In particular, the ester **5k** showed comparable activity with respect to that of compounds **4d** and **5d,** while its acid counterpart **4k** reported a twofold gain in activity shifting to low micromolar activity (**5k**, IC_50_ = 15.3 μM; **4k**, IC_50_ = 5.9 μM).

Following this trend, arylmethyloxy ether derivatives **4l–t** and **5l–t** reported the most promising inhibitory profile. Indeed, although the esters **5s,t** containing two aromatic rings in the 6-position of the quinolinone core were found inactive, the acid counterparts **4s,t** were found highly active (IC_50_ values 7.47 and 7.48 μM, respectively). This trend of activity could be ascribable to the coexistence of the carboxylic acid function in the 3-position, along with substituents characterized by both moderate degrees of freedom and steric hindrance in the 6-position of the quinolinonyl ring (a benzyloxybenzyl group in the case of derivative **4t** and a biphenylmethyl moiety for derivative **4s**).

Within this subseries, 7 derivatives (**4m,o,r–t** and **5l,o**) out of 18 tested showed high inhibitory activities, with IC_50_ values lower than 10 μM, 5 compounds (**4n,q** and **5m,p,r**) proved to be active with 10 μM < IC_50_ < 30, and only 6 ethers (**4l,p** and **5n,q,s,t**) reported inhibitory activity with IC_50_ values >30 μM.

Compounds **4q** and **5p** reported inhibitory potencies comparable to those of compounds **4d** and **5d** (**4q**, IC_50_ = 13.48 μM; **5p**, IC_50_ = 19.59 μM).

The ester **5o** and its acid counterpart **4o**, characterized by a methylnapht-1-yl group in position 6 of the quinolinonyl ring, proved to be the best acting compounds among the newly synthesized quinolinonyl derivatives showing IC_50_ values of 1.49 and 1.51 μM, respectively.

Interestingly, the regioisomers **4p** and **5p** of the compounds described above, obtained by replacing the methylnapht-1-yl group with a methylnapht-2-yl one, showed a decrease in activity by about 13–20 times.

A similar decrease in potency was obtained by substituting the methylnapht-1-yl group with a benzyl ring (**4l** and **5l**, IC_50_ = 34.0 and 8.0 μM, respectively), thus suggesting that for both acid and ester compounds, the methylnapht-1-yl group is advisable for enzymatic inhibition.

The dichloro derivatives **4m** and **5m**, isosters of compounds **4o** and **5o**, reported lower inhibitory potencies, as well. Indeed, the ester **5m** showed an IC_50_ value 16 times lower than its isoster **5o** (**5m**, IC_50_ = 24.03 μM; **5o**, IC_50_ = 1.49 μM). The decrease in activity was less evident for the corresponding acid **4m**, resulting in a 5-fold loss in activity with respect to its isosteric counterpart **4o** (**4m**, IC_50_ = 8.19 μM; **4o**, IC_50_ = 1.51 μM).

Differently, this trend of activity is not detected for the methylnapht-2-yl derivatives **6q**, **7q, 4p,** and **5p** and their dichloro isosters **4n** and **5n**, which showed comparable activity.

Likewise, no big difference in inhibitory potencies can be outlined between the dichloro derivatives **4n** and **5n** and the corresponding isomers **4m** and **5m**, with the sole exception of the acid 3,4-dichloro derivative **4m**, which is active at the micromolar concentration level (**4m**, IC_50_ = 8.19 μM). Finally, collectively, the arylmethyloxy acid derivatives resulted in more potent RHIs than the ester counterparts. Indeed, we obtained five acids active in the micromolar range, one compound around 10 μM, and three derivatives active around 30 μM concentrations. Only two of the corresponding esters showed IC_50_ values in the micromolar range, five compounds were active at concentrations higher that 20 μM, and two compounds were inactive up to 100 μM.

#### Cell-Based Assays

To determine the effect of compounds on viral replication, compounds **4d,k–t** and **5d,k–p,r** were chosen to test the antiviral activity in HeLa-CD4-LTR-β-gal cells (Table 2). In general, the acid derivatives gave measurable EC_50_ values that ranged from 1.73 to 16.1 μM with only compounds **4d,p,r** (out of 11 tested) inactive up to 50 μM. Among them, compound **4t** reported the lowest EC_50_ of the series, proving not to be cytotoxic up to high concentrations (100 μM), thus showing the best antiviral profile (SI > 57.8). In general, the ester derivatives showed a weaker activity when compared to the ones of the acid series, and only two compounds out of the eight tested were active in the low micromolar range (**5n**, IC_50_ = 14.6 μM; **5m**, IC_50_ = 17.8 μM).

**Table 2:** Biological Effects on RT-RDDP and IN Activities, Cytotoxicity, and Antiviral Activities of Compounds **4d,k–t** and **5d,k–p,r**

	activity in the enzyme assay IC_50_ (μM)^a^		antiviral activity (μM)		
cpd	RDDP^b^	IN^c^	EC_50_^d^	CC_50_^e^	SI^f^
**4d**	nt^g^	>100	>50	>200	
**4k**	>100	3.38 ± 0.42	5.4 ± 3.1	17.0 ± 4.0	3.1
**4l**	38.5 ± 7.1	0.41 ± 0.03	11.7 ± 2.5	>100	>8.6
**4m**	5.6 ± 0.6	>100	13.3 ± 4.5	>200	>15
**4n**	2.0 ± 0.8	3.25 ± 0.85	16.1 ± 5.5	>200	>12.4
**4o**	11.4 ± 2.6	>100	8.4 ± 0.7	51 ± 11	6.1
**4p**	nt	nt	>50	>200	
**4q**	5.1 ± 1.8	>100	11.3 ± 3.5	>200	>17.7
**4r**	nt	nt	>50	>200	
**4s**	11.6 ± 5.5	>250	2.5 ± 1.03	>100	>40
**4t**	2.2 ± 0.1	>100	1.73 ± 0.47	>100	>57.8
**5d**	nt	>100	>50	>200	
**5k**	nt	nt	>50	>100	
**5l**	nt	nt	>100	>100	
**5m**	1.88 ± 0.04	0.05 ± 0.01	17.8 ± 2.3	21.6 ± 2.8	1.2
**5n**	24.1 ± 8.6	9.45 ± 0.55	14.6 ± 3.1	20.6 ± 4.4	1.4
**5o**	nt	nt	>100	>100	
**5p**	nt	nt	>50	73 ± 24	
**5r**	nt	nt	>16	>200	
**11**		>100	2.9 ± 0.5	68 ± 10	>13.7
RAL^h^		0.019 ± 0.01			
EFV^i^	0.035 ± 0.011		0.53 ± 0.04		

a Inhibitory concentration 50% (μM) determined from dose-response curves.

b Experiments performed against HIV-1 RT-RDDP activity.

c Experiments performed against HIV-1 IN activity.

d Effective concentration 50% (μM).

e Cytotoxic concentration 50% (μM).

f Selectivity index = CC_50_/EC_50_.

g nt, not tested.

h RAL, raltegravir.

i EFV, efavirenz.

### Counter-Assays against IN and RDDP HIV-1 Activities

Since several RNase H active site inhibitors were reported to inhibit also other related viral targets;^22^ compounds **4e,k,l–o,r–t** and **5m,n** active against viral replication were tested for their ability to inhibit the other HIV-RT enzymatic function RDDP and the activity of the HIV-1 IN, structurally related to HIV-1 RNase H (Table 2).

The results showed that seven out of the 12 compared derivatives were selective for HIV-1 RNase H inhibition over HIV-1 IN inhibition, showing IC_50_ > 100 μM against the IN enzyme, three compounds showed comparable activities, and only two derivatives were substantially more active against IN than against RH.

Conversely, we did not observe a selectivity against the RT-RDDP activity since most of the tested compounds inhibited the polymerase function with IC_50_ values on the same order of magnitude of RNase H IC_50_ values. Here, we cannot exclude the possibility that the RDDP inhibition might result from an allosteric modulation of this RT function rather than a direct binding to the NNRTI binding site as already demonstrated for other RHIs.^52^ Nevertheless, this additional inhibition may contribute to the antiviral activity displayed in cell-based assays. Only two compounds showed selectivity for RH, namely, derivative **4k** that did not influence the RDDP activity up to 100 μM and **4o**. Interestingly, the last one was the best performing RNase H inhibitor, with an increase of 7.5-fold against RDDP (1.51 vs 11.4 μM) and was totally inactive against HIV-1 IN (IC_50_ > 100 μM). It was chosen to be further characterized for its binding mode, together with compound **4t**, which showed the most promising antiviral activity.

### Molecular Modeling

**Figure 3:**
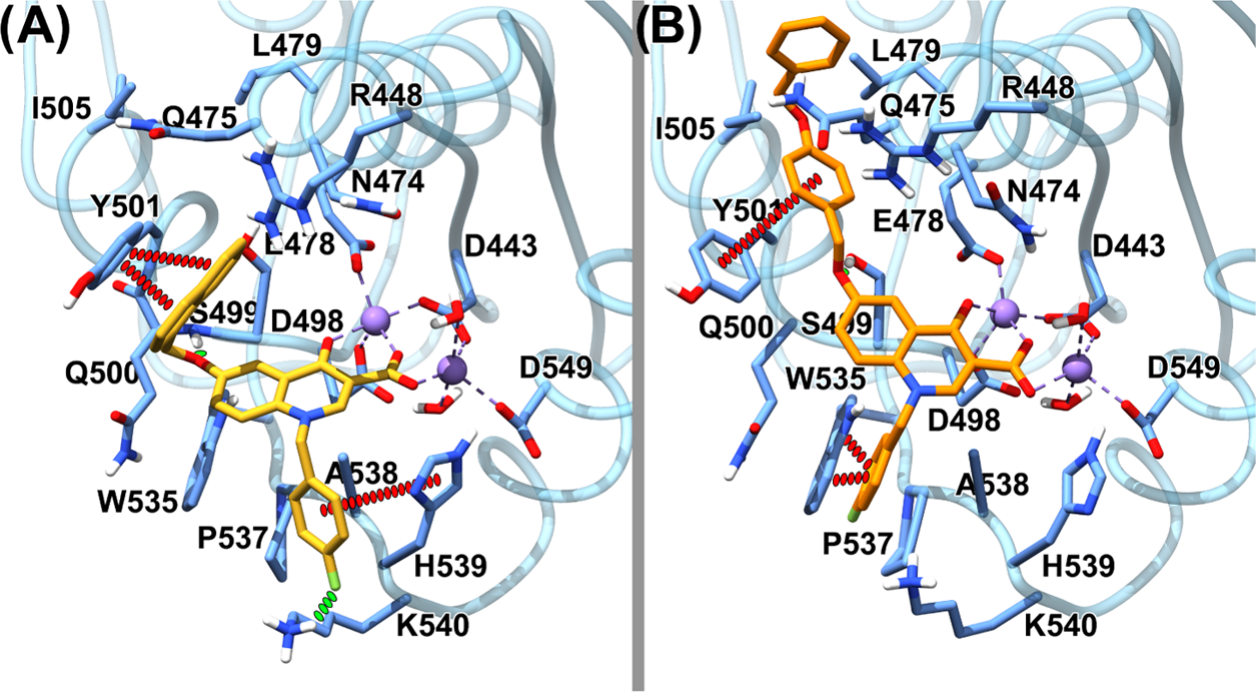
Predicted binding poses of **4o** (A) and **4t** (B) in the HIV RNase H binding site (PDB 3QIP). Important residues are labeled. **4o** is represented as yellow sticks, **4t** as orange sticks, the magnesium ions are depicted in purple, and their coordination with the nearby atoms is also represented in purple. H-bonds are depicted as dashed green lines. Charge-transfer interactions are represented as dashed red lines. The protein is depicted as blue ribbons and sticks.

**Figure 4:**
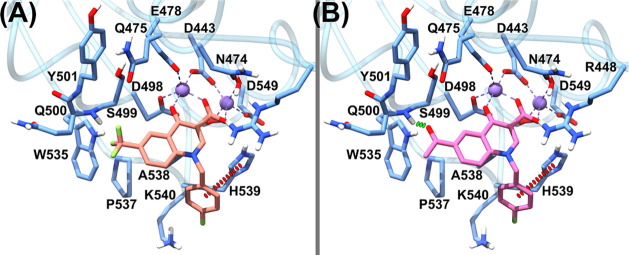
Predicted binding poses of **4c** (A) and **4d** (B) in the HIV RNase H binding site (PDB 3QIP). Important residues are labeled. **4c** is represented as salmon sticks, **4t** as magenta sticks, the magnesium ions are depicted in purple, and their coordination with the nearby atoms is also represented in purple. H-bonds are depicted as dashed green lines. Charge-transfer interactions are represented as dashed red lines. The protein is depicted as blue ribbons and sticks.

To clarify the reasons behind the inhibitory activity displayed by the novel compounds, molecular docking studies were performed on **4o** and **4t**. In particular, to predict the binding poses of compounds **4o** and **4t** in the RNase H binding site (X-ray crystal structure with the PDB code 3QIP)^53^ and to adequately probe the possible conformational changes in the active site induced by the rather bulky side chains at the 6-position of the quinolinonyl core, we elected to employ the Induced Fit routine of Glide docking software^54,55^ (see Molecular Modeling methods). In general, we expect that the whole set of newly designed inhibitors would bind to the viral enzyme with similar poses to those that we are reporting for **4o** (Figure 3A) and **4t** (Figure 3B).

Our model suggests for both **4o** and **4t** that the oxygen atoms of the ketone and the position-4 carboxyl/ester moiety tightly chelate the Mg^2+^ atoms in the active site, in a geometry that is consistent with other cocrystallized HIV-1 RNase H inhibitors.^53,56^

Interestingly, the 4-carboxyl group is localized in a highly polar section of the binding site, which comprises H539, D549, D443, D498, and E478 and two conserved water molecules that take part in the chelation of one of the magnesium ions. While we infer that the ester derivatives **5a–t** should engage in the same interaction pattern that characterizes their acidic counterparts, our theoretical model would place the esters’ lipophilic ethyl chain toward the above-described highly hydrophilic area. In some cases, this should unfavorably impact the binding affinity.

The quinolinonyl core of **4o** forms van der Waals contacts with the side chains of W535 and A538. In the **4t** case, the said core adopts a slightly different pose, possibly diminishing the strength of the van der Waals contacts with the two residues. The **4o** pendant *p*-fluorobenzyl ring is lodged in a cleft lined by the side chains of the polar residues H539 and K540. Here, the phenyl ring establishes a T-shaped charge-transfer interaction with H539, which is intensified by the electron-withdrawing (EWG) fluorine substituent and a H-bonding interaction between its *p*-fluorine atom and the K540 side chain.

Regarding the **4t** pendant *p*-fluorobenzyl ring, while it is predicted to point toward the same cleft, it would be oriented away from H539 and closer to the side chains of P537 and W535, possibly giving rise to a parallel-displaced π–π interaction with the latter residue, also enhanced by the *p*-fluorine atom. Besides, the K540 side chain would be oriented to form a cation-π with the ligand pendant phenyl ring, although this interaction should be weakened by the presence of an EWG substituent such as fluorine.^57,58^

As for the 6-position substituent, several of the newly designed compounds feature an oxygen atom, which should be well-positioned to accept a H-bond from either the backbone NH of Q500, as exemplified by the **4o** binding pose, or the side chain of the nearby S499, as in the **4t** case. Furthermore, the **4o** naphthylmethoxy group would be positioned in a polar pocket lined by the residues R448, N474, Q475, S499, and Y501. Here, like the other arylmethyloxy ether derivatives, **4o** naphthyl can form a parallel-displaced charge-transfer interaction with the aromatic side chain of Y501, similar to what we already demonstrated for another class of RNase H inhibitors.^36^ On the other hand, our *in silico* analysis also revealed that the compound **4t** methylphenyl-4-oxybenzyl group should reach an enzyme region that is only partially overlapping with the binding site area contacted by the naphthylmethoxy moiety of **4o**. While the **4t** extended chain should still engage in a π–π interaction with Y501 through its aromatic portions, it should also be able to access an adjacent cavity lined by the residues Q475, L479, Y501, and I505, providing further anchoring points for the ligand. It could also be inferred that the other compounds with a comparably long 6-position substituent, such as **4r** and **4s**, might lodge their chain in the same pocket contacted by **4t**. To better elucidate the interaction pattern established by derivatives devoid of aromatic moieties at the 6-position (**4a**–**h**,**j** and **5a**–**h**,**j**), we also docked compounds **4c** and **4d** in the RNase H binding site. The resulting docking solutions are presented in Figure 4. Indeed, with the exception of the missing contacts with Y501, the predicted binding poses for the two ligands are largely congruent with what we detailed for **4o**. Here, the **4d** carbonyl oxygen of the acetyl group at the 6-position seems to be favorably positioned to accept a H-bond from the backbone NH of Q500. However, in **4c**, the trifluoride substituent would establish a weak H-bond with the Q500 backbone NH. Thus, it could be inferred that only some substituents, such as the acetyl and the nitro group in **4d** and **4e**, respectively, possess a spatial arrangement that can tightly engage in this specific contact.

Arguably, the described interacting points (*i.e.*, a strong H-bond-accepting atom and/or an aromatic ring) in position 6 of the quinolone core should provide anchoring points that help in stabilizing the chelation geometry. Thus, these considerations could account for the weaker activity, or complete lack thereof, of the derivatives **4a–c,f–h** and **5a–c,f–h**.

### Site-Directed Mutagenesis

**Figure 5:**
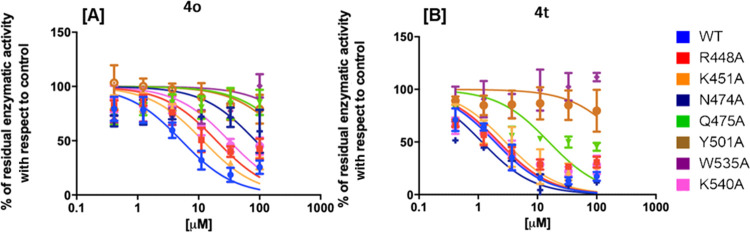
Inhibition of HIV-1 RT-associated RNase H activity of mutated HIV-1 RTs by quinolinonyl non-diketo acid derivatives. Panel A: **4o**; panel B: **4t**.

To experimentally verify the binding model suggested by computational studies, compounds **4o** and **4t** were tested against the RNase H activity of several point-mutants of HIV-1 RNase H, generated by independently introducing an alanine substitution at residues R448, K451, N474, Q475, Y501, W535, and K540 (Figure 5). An activity curve was performed for all of the enzymes (Figure S1), and a concentration was chosen in the linear dose-response range to perform the enzymatic-inhibition assays. In agreement with the proposed binding pose, results showed that the potency of inhibition of compound **4o** was significantly affected when tested against all of the mutated enzymes (Figure 5A) with a loss of potency of 3.7-fold against R448A, 2.2-fold against K451A, 6.0-fold against K540A, 16.3-fold against N474A, and completely losing inhibitory activity against the mutants Q475A, Y501A, and W535A, with a loss of potency greater than 19.1-fold (see the Supporting Information Table S1). According to the binding model, results showed that compound **4t** binds in a slightly different orientation, establishing a less extended network of interactions: its inhibitory activity was not affected by the presence of R448A, K451A, N474A, or K540A substitutions (Figure 5A) (*p* value > 0.05; see the Supporting Information Table S2), while it lost 8.2-fold potency against Q475A RT and was totally inactive against the RNase H function of Y501A and W535A RTs, with a decrease in potency greater than >52.4-fold. Interestingly, the analyzed residues are among the most conserved toward naïve and treated patients,^15^ with a degree of conservation up to 99.9% for Y501, N474, and Y501 and greater than 100% for W535 and Q475, thereby increasing the evidence that the design of inhibitors targeting conserved regions within the RNase H active site is a possible path for lead development.

### Investigation of Magnesium Complexation

**Figure 6:**
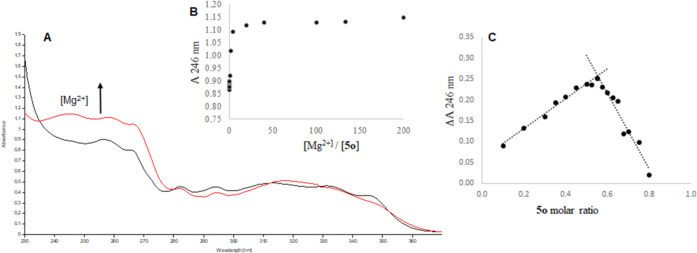
[A] UV spectra of **5o** in EtOH 3.81 10^–5^ M (black trace) and **5o** (3.81 10^–5^ M) + MgCl_2_ (3.81 10^–3^ M) (red trace). [B] Increments of A at 246 nm obtained during the titration of **5o** with MgCl_2_. [C] Job’s plot obtained for **5o** and MgCl_2_. Δ*A* at 246 nm was plotted *vs* the molar ratio of **5o**. The maximum ΔA was observed at *X* = 0.54, which corresponds to a stoichiometry of 1:1 for the complex **5o**–Mg^2+^.

To investigate the potential importance of the interaction between the active compounds and Mg^2+^ ions, spectrophotometric complexation studies were carried out on the best active derivatives **4o** and **5o**. Titration of compound **5o** with MgCl_2_ produced an increase in absorbance at 246 nm (Figure 6 panel B), thereby indicating coordination of this compound with Mg^2+^. Moreover, as observed in Job’s plot (Figure 6, panel C), the stoichiometry of the complex **5o**–Mg^2+^ is 1:1. Similarly, for acid **4o**, an increase in absorbance was observed at 256 nm (see the Supporting Information Figure S2 panel B), supporting our hypothesis according to which the shortening of the DKA branch into a carboxylic acid function could, together with the ketone group of the quinolinone ring, interact with the Mg^2+^ ions. Also for derivative **4o**, we observed a stoichiometry of 1:1 for the complex **4o**–Mg^2+^(see the Supporting Information Figure S1 panel C).

## Conclusions

In this work, we reported a new series of quinolonyl non-DKA derivatives as RHIs. This new class of compounds was conceived by shortening the quinolonyl DKA chain typical of INSTI into a carboxylic acid function that, together with the ketone group in the 4-position of the quinolinone ring, could still chelate the two Mg^2+^ ions. The newly designed compounds showed activity against RH and were also confirmed to be able to chelate the magnesium ions by spectrophotometric complexation studies.

Among the newly synthesized derivatives, arylmethyloxy acid quinolonyl derivatives demonstrated inhibitory activities within the micromolar/low micromolar range, resulting in IC_50_ values lower than that of the ester counterparts, with compound **4o** being the most potent acid derivative (IC_50_ = 1.51 μM). Docking studies within the RNase H catalytic site highlighted a possible reason for this trend of activity. Indeed, the 4-carboxyl group is localized in a highly polar section of the binding site. As a result, the esters would place their lipophilic ethyl chain toward this highly hydrophilic area and this should unfavorably impact the binding affinity. Interestingly, this trend was also observed in acutely infected cells, with derivative **4t** being the best acting compound (EC_50_ = 1.73 μM) with no cytotoxic activity. Site-directed mutagenesis experiments confirmed the docking calculations, demonstrating also that our compounds are capable of interacting with amino acids highly conserved among naïve and treated patients. Overall, these results confirmed the effectiveness of this class of quinolonyl non-DKA derivatives as new RHIs.

It is worth noting that the quinolonyl DKAs were in general active at low nanomolar concentrations against IN, showing marginal activity against RH. Conversely, the new quinolonyl non-DKAs were good RH inhibitors, with marginal activity against IN, as hypothesized in the rationale. This series was nonselective against RDDP of the RT so that the antiviral activity that resulted can be ascribed to inhibiting effects of both RT functions.

## Experimental Section

### Chemistry

#### General

Melting points were determined on a Bobby Stuart Scientific SMP1 melting point apparatus and are uncorrected. Compound purity was always >95% as determined by combustion analysis. Analytical results agreed to within ±0.40% of the theoretical values. IR spectra were recorded on a PerkinElmer Spectrum-One spectrophotometer. ^1^H NMR spectra were recorded at 400 MHz on a Bruker AC 400 Ultrashield 10 spectrophotometer (400 MHz). Dimethyl sulfoxide-*d*_6_ 99.9% (CAS 2206–27–1), deuterochloroform 98.8% (CAS 865–49–6), and acetone-*d*_6_ 99.9% (CAS 666–52–4) of isotopic purity (Aldrich) were used. Column chromatographies were performed on silica gel (Merck; 70–230 mesh). All compounds were routinely checked on thin-layer chromatography (TLC) using aluminum-baked silica gel plates (Fluka DC-Alufolien Kieselgel 60 F_254_). Developed plates were visualized by UV light. Solvents were of reagent grade and, when necessary, were purified and dried by standard methods. Concentration of solutions after reactions and extractions involved the use of a rotary evaporator (Büchi) operating at a reduced pressure (ca. 20 Torr). Organic solutions were dried over anhydrous sodium sulfate (Merck). All solvents were freshly distilled under nitrogen and stored over molecular sieves for at least 3 h prior to use. Analytical results agreed to within ±0.40% of the theoretical values.

#### General Experimental Procedures

##### General Procedure A (GP-A) to Obtain *O*-Alkyl Derivatives **10a–j**

To a mixture of diethyl 2-(((4-hydroxyphenyl)amino)methylene)malonate (17.9 mmol) in anhydrous DMF (80 mL), *t*-BuOK (26.9 mmol) and the proper halide (26.9 mmol) were added at 0 °C. Then, the resulting mixture was stirred at room temperature for the proper time and monitored by TLC.

The reaction was quenched with water (30 mL) and extracted with ethyl acetate (3 × 50 mL). The combined organic layers were washed with brine (200 mL), dried over Na_2_SO_4_, concentrated, and purified by column chromatography on silica gel. For derivate **10a**, the alkylating agent was obtained as a free base, upon the reaction between the commercially available 3-dimethylamino-1-propyl chloride hydrochloride and triethylamine in anhydrous THF. For each compound, alkylating agent; reaction time; chromatography eluent; recrystallization solvent; yield (%); melting point (°C); IR; ^1^H NMR; and elemental analysis are reported.

##### General Procedure B (GP-B) to Obtain Quinolinonyl Ester Derivatives (**8b,k,j,l–t**)

The proper substituted anilidomethylenemalonic ester (19.0 mmol) was suspended in diphenyl ether (0.304 mol, 48 mL), stirred under reflux for the proper time, and monitored by TLC. Upon completion of the reaction, the mixture was cooled down and poured into *n*-hexane (50 mL). The resulting precipitate was filtered, washed three times with *n*-hexane (10 mL) and petroleum ether, and dried under an IR lamp to afford the pure product. For each compound, reaction time; recrystallization solvent; yield (%); melting point (°C); IR; ^1^H NMR; and elemental analysis are reported.

##### General Procedure C (GP-C) to Obtain *N*-Alkyl Quinolinonyl Ester Derivatives (**5a–t**)

A mixture of the appropriate quinolinonyl ester (15.0 mmol), 4-fluorobenzyl bromide (45.0 mol), and K_2_CO_3_ anhydrous (21.0 mmol) in DMF anhydrous (130 mL) was stirred at 100 °C for the proper time and monitored by TLC. The mixture was cooled, treated with water (40 mL), and extracted with ethyl acetate (3 × 100 mL). The organic layer was washed with brine (200 mL), dried over anhydrous sodium sulfate, and concentrated under vacuum. The crude product was purified by chromatography (SiO_2_) to afford the pure product. For each compound, reaction time; chromatography eluent; recrystallization solvent; yield (%); melting point (°C); IR; ^1^H NMR; and elemental analysis are reported.

##### General Procedure D (GP-D) to Obtain Quinolinonyl Carboxylic Acid Derivatives (**4a–t**)

A solution of NaOH 20% (0.172 mol) in distilled water was added to a suspension of the appropriate ester (0.010 mol) in 1:1 THF/ethanol (50 mL), and the reaction was stirred vigorously under reflux for the proper time. The reaction was monitored by TLC. Upon completion of the reaction, the organic phase was removed under vacuum and the resulting suspension was acidified with 1 N HCl (pH 4–5). The resulting solid was filtered, washed with water, and dried under an IR lamp to afford the product of interest. For **4b**, 0.4 M LiOH (50.0 mmol) was used as a base instead of NaOH and the reaction was performed at room temperature. For each compound, reaction time; recrystallization solvent; yield (%); melting point (°C); IR; ^1^H NMR; and elemental analysis are reported.

##### 1-(4-Fluorobenzyl)-4-oxo-1,4-dihydroquinoline-3-carboxylic Acid (**4a**)

Synthesis, analytical, and spectroscopic data are reported in the literature.^41^

##### 6-Cyano-1-(4-fluorobenzyl)-4-oxo-1,4-dihydroquinoline-3-carboxylic Acid (**4b**)

Compound **4b** was prepared from ethyl 6-cyano-1-(4-fluorobenzyl)-4-oxo-1,4-dihydroquinoline-3-carboxylate by means of GP-D; 4 h; ethanol; 80% as a yellow solid; 247 °C; IR ν 1604 (C=O), 1717 (C=O), 2232 (CN), 3049 (COOH) cm^–1^; ^1^H NMR (400 MHz DMSO-*d*_6_, δ) 5.87 (s, 2H, CH_2_), 7.16–7.21 (m, 2H, benzene H), 7.35–7.39 (m, 2H, benzene H), 8.01 (d, *J* = 8.5 Hz, 2H, quinolinone H), 8.23 (d, *J* = 8.5 Hz, 2H, quinolinone H), 8.75 (s, 1H, quinolinone H), 9.33 (s, 1H, quinolinone H), 14.5 (br s, 1H, COOH). Anal. calcd for C_18_H_11_FN_2_O_3_: C, 67.08; H, 3.44; F, 5.89; N, 8.69%. Found: C, 67.22; H, 3.45; F, 5.91; N, 8.70%.

##### 1-(4-Fluorobenzyl)-4-oxo-6-(trifluoromethyl)-1,4-dihydroquinoline-3-carboxylic Acid (**4c**)

Compound **4c** was prepared from ethyl 1-(4-fluorobenzyl)-4-oxo-6-(trifluoromethyl)-1,4-dihydroquinoline-3-carboxylate by means of GP-D; 1 h; ethanol; 100% as a white solid; >300 °C; IR ν 1612 (C=O), 1706 (C=O), 3078 (COOH) cm^–1^; ^1^H NMR (400 MHz DMSO-*d*_6_, δ) 6.00 (s, 2H, CH_2_), 7.14–7.19 (m, 2H, benzene H), 7.47–7.50 (m, 2H, benzene H), 8.13–8.15 (m, 2H, quinolinone H), 8.72 (s, 1H, quinolinone H), 9.19 (s, 1H quinolinone H), 14.38 (br s, 1H, COOH). Anal. calcd for C_18_H_11_F_4_NO_3_: C, 59.19; H, 3.04; F, 20.80; N, 3.83%. Found: C, 59.39; H, 3.03; F, 20.82; N, 3.84%.

##### 6-Acetyl-1-(4-fluorobenzyl)-4-oxo-1,4-dihydroquinoline-3-carboxylic Acid (**4d**)

Synthesis, analytical, and spectroscopic data are reported in the literature.^38^

##### 1-(4-Fluorobenzyl)-6-nitro-4-oxo-1,4-dihydroquinoline-3-carboxylic Acid (**4e**)

Compound **4e** was prepared from ethyl 1-(4-fluorobenzyl)-6-nitro-4-oxo-1,4-dihydroquinoline-3-carboxylate by means of GP-D; 1 h; ethanol; 93% as a yellow solid; 256 °C; IR ν 1454 (NO_2_), 1617 (C=O), 1719 (C=O) cm^–1^, 3070 (COOH); ^1^H NMR (400 MHz DMSO-*d*_6_, δ) 5.89 (s, 2H, CH_2_), 7.18–7.22 (m, 2H, benzene H), 7.33–7.38 (m, 2H, benzene H), 8.08 (d, *J* = 8.5 Hz, 1H, quinolinone H), 8.58 (d, *J* = 8.5 Hz, 1H, quinolinone H), 9.02 (s, 1H, quinolinone H), 9.36 (s, 1H, quinolinone H), 14.35 (br s, 1H, COOH). Anal. calcd for C_17_H_11_FN_2_O_5_: C, 59.65; H, 3.24; F, 5.55; N, 8.18%. Found: C, 59.72; H, 3.25; F, 5.56; N, 8.20%.

##### 1-(4-Fluorobenzyl)-6-(methylsulfonyl)-4-oxo-1,4-dihydroquinoline-3-carboxylic Acid (**4f**)

Compound **4f** was prepared from ethyl 1-(4-fluorobenzyl)-6-(methylsulfonyl)-4-oxo-1,4-dihydroquinoline-3-carboxylate by means of GP-D; 2 h; methanol; 87% as a white solid; 254 °C; IR ν 1033 (SO_2_CH_3_), 1622 (C=O), 1736 (C=O), 3680 (COOH) cm^–1^; ^1^H NMR (400 MHz DMSO-*d*_6_, δ) 3.35 (s, 3H, CH_3_), 5.88 (s, 2H, CH_2_), 7.18 (t, 2H, benzene H), 7.35–7.39 (m, 2H, benzene H), 8.08 (d, *J* = 8.5 Hz, 1H, quinolinone H), 8.29 (s, *J* = 8.5 Hz, 1H, quinolinone H), 8.81 (s, 1H, quinolinone H), 9.36 (s, 1H, quinolinone H), 14.56 (s, 1H, COOH). Anal. calcd for C_18_H_14_FNO_5_S: C, 57.60; H, 3.76; F, 5.06; N, 3.73; S, 8.54%. Found: C, 57.72; H, 3.77; F, 5.08; N, 3.72; S, 8.55%.

##### 1-(4-Fluorobenzyl)-6-hydroxy-4-oxo-1,4-dihydroquinoline-3-carboxylic Acid (**4g**)

Compound **4g** was prepared from ethyl 1-(4-fluorobenzyl)-6-hydroxy-4-oxo-1,4-dihydroquinoline-3-carboxylate by means of GP-D; 1 h; methanol; 89% as a white solid; >300 °C; IR ν 1619 (C=O), 1718 (C=O), 3045 (COOH) cm^–1^; ^1^H NMR (400 MHz DMSO-*d*_6_, δ) 5.82 (s, 2H, CH_2_), 7.17–7.21 (m, 2H, benzene H), 7.32–7.36 (m, 3H, benzene H and quinolone H), 7.66 (s, 1H, quinolinone H), 7.77 (d, *J* = 8.5 Hz, 1H, quinolinone H), 9.17 (s, 1H, quinolinone H), 10.41 (s, 1H, OH), 15.35 (s, 1H, COOH). Anal. calcd for C_17_H_12_FNO_4_: C, 65.18; H, 3.86; F, 6.06; N, 4.47%. Found: C, 65.36; H, 3.87; F, 6.05; N, 4.46%.

##### 1-(4-Fluorobenzyl)-6-methoxy-4-oxo-1,4-dihydroquinoline-3-carboxylic Acid (**4h**)

Compound **4h** was prepared from ethyl 1-(4-fluorobenzyl)-6-methoxy-4-oxo-1,4-dihydroquinoline-3-carboxylate by means of GP-D; 2 h; ethanol; 98% as a white solid; >300 °C; IR ν 1609 (C=O), 1700 (C=O), 3091 (COOH) cm^–1^; ^1^H NMR (400 MHz DMSO-*d*_6_, δ) 3.90 (s, 3H, −OCH_3_), 5.86 (s, 2H, CH_2_), 7.17–7.21 (m, 2H, benzene H), 7.32–7.33 (m, 2H, benzene H), 7.51 (d, *J* = 8.5 Hz, 1H, quinolinone H), 7.75 (s, 1H, quinolinone H), 7.84 (d, *J* = 8.5 Hz, 1H, quinolinone H), 9.23 (s, 1H, quinolinone), 15.33 (br s, 1H, COOH). Anal. calcd for C_18_H_14_FNO_4_: C, 66.05; H, 4.31; F, 5.80; N, 4.28%. Found: C, 66.17; H, 4.32; F, 5.81; N, 4.29%.

##### 1-(4-Fluorobenzyl)-4-oxo-6-phenoxy-1,4-dihydroquinoline-3-carboxylic Acid (**4i**)

Compound **4i** was prepared from ethyl 1-(4-fluorobenzyl)-4-oxo-6-phenoxy-1,4-dihydroquinoline-3-carboxylate by means of GP-D; 2 h; THF; 95% as a white solid; 274 °C; IR ν 1617 (C=O), 1707 (C=O), 3059 (COOH) cm^–1^; ^1^H NMR (400 MHz DMSO-*d*_6_, δ) 5.84 (s, 2H, CH_2_), 7.14–7.21 (m, 4H, benzene H, and quinolinone H), 7.27 (t, *J* = 8.0 Hz, 1H, benzene H), 7.28–7.49 (m, 4H, benzene H), 7.62–7.64 (m, 2H, benzene H and quinolinone H), 7.93 (d, *J* = 8.5 Hz, 1H, quinolinone H), 9.21 (s, 1H, quinolone H), 15.00 (br s, 1H, COOH). Anal. calcd for C_23_H_16_FNO_4_: C, 70.95; H, 4.14; F, 4.88; N, 3.60%. Found: C, 71.08; H, 4.15; F, 4.89; N, 3.59%.

##### 6-(3-(Dimethylamino)propoxy)-1-(4-fluorobenzyl)-4-oxo-1,4-dihydroquinoline-3-carboxylic Acid (**4j**)

Compound **4j** was prepared from ethyl 6-(3-(dimethylamino)propoxy)-1-(4-fluorobenzyl)-4-oxo-1,4-dihydroquinoline-3-carboxylate by means of GP-D; 1 h; ethanol; 87% as a brown solid; >300 °C; IR v 1554 (C=O) and 1702 (C=O), 3105 (OH) cm^–1^; ^1^H NMR (400 MHz DMSO-*d*_6_, δ) 2.04 (t, *J* = 7.0 Hz, 2H, CH_2_), 2.40 (s, 3H, CH_3_), 2.42 (s, 3H, CH_3_), 2.81–2.86 (m, 2H, CH_2_), 4.18 (t, *J* = 7.0 Hz, 2H, CH_2_), 5.86 (s, 2H, CH_2_), 7.17–7.19 (m, 2H, benzene H), 7.21–7.23 (m, 2H, benzene H), 7.34 (d, *J* = 8.5 Hz, 1H, quinolinone H), 7.75 (s, 1H, quinolinone H), 7.83–7.85 (d, *J* = 8.5 Hz, 1H, quinolinone H), 9.23 (s, 1H, quinolinone), 15.30 (br s, 1H, COOH). Anal. calcd for C_22_H_23_FN_2_O_4_: C, 66.32; H, 5.82; F, 4.77; N, 7.03%. Found: C, 66.40; H, 5.83; F, 4.76; N, 7.02%.

##### (*E*)-1-(4-Fluorobenzyl)-4-oxo-6-(3-oxo-3-phenylprop-1-en-1-yl)-1,4-dihydroquinoline-3 carboxylic Acid (**4k**)

Compound **4k** was prepared from (*E*)-ethyl 1-(4-fluorobenzyl)-4-oxo-6-(3-oxo-3-phenylprop-1-en-1-yl)-1,4-dihydroquinoline-3-carboxylate by means of GP-D; 1 h; ethanol; 86% as a white solid; IR ν 1599 (C=O), 1623 (C=O), 1655 (C=O), 3361 (COOH) cm^–1^; ^1^H NMR (400 MHz DMSO-*d*_6_, δ) 5.82 (s, 2H, CH_2_), 7.12–7.20 (m, 2H, benzene H), 7.32–7.36 (m, 2H, benzene H), 7.54–7.59 (m, 3H, benzene H, and alkene H), 7.79–7.83 (m, 2H, benzene H), 8.01–8.10 (m, 3H, benzene H, and alkene H), 8.67 (s, 1H, quinolinone H), 9.22 (s, 1H, quinolinone H), 14,95 (s, 1H, COOH). Anal. calcd for C_26_H_18_FNO_4_: C, 73.06; H, 4.24; F, 4.44; N, 3.28%. Found: C, 73.15; H, 4.25; F, 4.43; N, 3.29%.

##### 6-(Benzyloxy)-1-(4-fluorobenzyl)-4-oxo-1,4-dihydroquinoline-3-carboxylic Acid (**4l**)

Compound **4l** was prepared from ethyl 6-(benzyloxy)-1-(4-fluorobenzyl)-4-oxo-1,4-dihydroquinoline-3-carboxylate by means of GP-D; 1.5 h; ethanol; 89% as a white solid; 248 °C; IR ν 1616 (C=O), 1711 (C=O), 3073 (OH) cm^–1^; ^1^H NMR (400 MHz DMSO-*d*_6_, δ) 5.22 (s, 2H, CH_2_), 5.83 (s, 2H, CH_2_), 7.17–7.21 (m, 4H, benzene H), 7.31–7.33 (m, 2H, benzene H), 7.51–55 (m, 4H, benzene H, and quinolinone H), 7.81 (m, 2H, benzene H, and quinolinone H), 9.20 (d, 1H, *J* = 8.5 Hz, quinolinone H), 15.30 (br s, 1H, COOH). Anal. calcd for C_24_H_18_FNO_4_: C, 71.46; H, 4.50; F, 4.71; N, 3.47%. Found: C, 71.35; H, 4.49; F, 4.70; N, 3.48%.

##### 6-((2,3-Dichlorobenzyl)oxy)-1-(4-fluorobenzyl)-4-oxo-1,4-dihydroquinoline-3-carboxylic Acid (**4m**)

Compound **4m** was prepared from ethyl 6-((2,3-dichlorobenzyl)oxy)-1-(4-fluorobenzyl)-4-oxo-1,4-dihydroquinoline-3-carboxylate by means of GP-D; 2 h; toluene; 72% as a white solid; 263 °C; IR ν 1615 (C=O), 1702 (C=O), 3063 (COOH) cm^–1^; ^1^H NMR (400 MHz DMSO-*d*_6_, δ) 5.37 (s, 2H, CH_2_), 5.86 (s, 2H, CH_2_), 7.17–7.22 (m, 2H, benzene H), 7.33–7.37 (m, 2H, benzene H), 7.41–7.45 (m, 1H, benzene H), 7.59–7.62 (m, 2H, benzene H, and quinolinone H), 7.68 (d, *J* = 8.5 Hz, 1H, benzene H), 7.85–7.89 (m, 2H, quinolinone H), 9.24 (s, 1H, quinolinone H), 15.25 (s, 1H, COOH). Anal. calcd for C_24_H_16_Cl_2_FNO_4_: C, 61.03; H, 3.41; Cl, 15.01; F, 4.02; N, 2.97%. Found: C, 61.13; H, 3.42; Cl, 15.05; F, 4.01; N, 2.98%.

##### 6-((3,4-Dichlorobenzyl)oxy)-1-(4-fluorobenzyl)-4-oxo-1,4-dihydroquinoline-3-carboxylic Acid (**4n**)

Compound **4n** was prepared from ethyl 6-((3,4-dichlorobenzyl)oxy)-1-(4-fluorobenzyl)-4-oxo-1,4-dihydroquinoline-3-carboxylate by means of GP-D; 1 h; toluene; 73% as a white solid; 260 °C; IR ν 1626 (C=O), 1705 (C=O), 3675 (COOH) cm^–1^; ^1^H NMR (400 MHz DMSO-*d*_6_, δ) 5.29 (s, 2H, CH_2_), 5.86 (s, 2H, CH_2_), 7.17–7.20 (m, 2H, benzene H), 7.32–7.36 (m, 2H, benzene H), 7.49 (d, *J* = 8.5 Hz, 1H, quinolinone H), 7.55 (d, *J* = 8.0 Hz, 1H, benzene H), 7.60 (d, *J* = 8.5 Hz, 1H, quinolinone H), 7.67 (s, 1H, benzene H), 7.69–7.88 (m, 2H, benzene H, and quinolinone H), 9.23 (s, 1H, quinolinone H), 15.27 (s, 1H, COOH). Anal. calcd for C_24_H_16_Cl_2_FNO_4_: C, 61.03; H, 3.41; Cl, 15.01; F, 4.02; N, 2.97%. Found: C, 60.98; H, 3.39; Cl, 15.00; F, 4.03; N, 2.96%.

##### 1-(4-Fluorobenzyl)-6-(naphthalen-1-ylmethoxy)-4-oxo-1,4-dihydroquinoline-3-carboxylic Acid (**4o**)

Compound **4o** was prepared from ethyl 1-(4-fluorobenzyl)-6-(naphthalen-1-ylmethoxy)-4-oxo-1,4-dihydroquinoline-3-carboxylate by means of GP-D; 2 h; toluene; 100% as an orange solid; 251 °C; IR ν 1598 (C=O), 1730 (C=O), 3081 (COOH) cm^–1^; ^1^H NMR (400 MHz DMSO-*d*_6_, δ) 5.72 (s, 2H, CH_2_), 5.86 (s, 2H, CH_2_), 7.16–7.21 (m, 2H, benzene H), 7.33–7.36 (m, 2H, benzene H), 7.50–7.61 (m, 4H, naphthalene H), 7.70 (d, *J* = 8.7 Hz, 1H, quinolinone H), 7.86 (d, *J* = 8.5 Hz, 1H, quinolinone H), 7.94–7.99 (m, 3H, naphthalene H), 8.10 (s, 1H, quinolinone H), 9.15 (s, 1H, quinolinone H), 15.22 (br s, 1H, COOH). Anal. calcd for C_28_H_20_FNO_4_: C, 74.16; H, 4.45; F, 4.19; N, 3.09%. Found: C, 74.21; H, 4.44; F, 4.21; N, 3.10%.

##### 1-(4-Fluorobenzyl)-6-(naphthalen-2-ylmethoxy)-4-oxo-1,4-dihydroquinoline-3-carboxylic Acid (**4p**)

Compound **4p** was prepared from ethyl 1-(4-fluorobenzyl)-6-(naphthalen-2-ylmethoxy)-4-oxo-1,4-dihydroquinoline-3-carboxylate by means of GP-D; 1 h; toluene; 68% as a white solid; 296 °C; IR ν 1613 (C=O), 1702 (C=O), 3660 (COOH) cm^–1^; ^1^H NMR (400 MHz DMSO-*d*_6_, δ) 5.36 (s, 2H, CH_2_), 5.76 (s, 2H, CH_2_), 7.08–7.17 (m, 2H, benzene H), 7.25–7.27 (m, 2H, benzene H), 7.43–7.45 (m, 3H, naphthalene H), 7.48–7.51 (m, 2H, naphthalene H, and quinolinone H), 7.55–7.88 (m, 5H, naphthalene H, and quinolinone H), 7.93 (s, 1H, quinolinone H), 9.13 (s, 1H, quinolinone H), 15.18 (s, 1H, COOH). Anal. calcd for C_28_H_20_FNO_4_: C, 74.16; H, 4.45; F, 4.19; N, 3.09%. Found: C, 74.22; H, 4.44; F, 4.21; N, 3.11%.

##### 6-(Benzo[*d*][1,3]dioxol-5-ylmethoxy)-1-(4-fluorobenzyl)-4-oxo-1,4-dihydroquinoline-3-carboxylic Acid (**4q**)

Compound **4q** was prepared from ethyl 6-(benzo[*d*][1,3]dioxol-5-ylmethoxy)-1-(4-fluorobenzyl)-4-oxo-1,4-dihydroquinoline-3-carboxylate by means of GP-D; 2 h; methanol; 50% as a white solid; 289 °C; IR ν 1626 (C=O), 1715 (C=O), 3357 (COOH) cm^–1^; ^1^H NMR (400 MHz DMSO-*d*_6_, δ) 5.09 (s, 2H, CH_2_), 5.61 (s, 2H, CH_2_), 5.99 (s, 2H, CH_2_), 6.86–6.97 (m, 2H, benzodioxolane H), 7.02 (s, 1H, benzodioxolane H), 7.15–7.18 (m, 2H, benzene H), 7.19–7.25 (m, 2H, benzene H), 7.27 (d, *J* = 8.5 Hz, 1H, quinolinone H), 7.42 (d, *J* = 8.5 Hz, 1H, quinolone H), 7.82 (s, 1H, quinolone H), 8.98 (s, 1H, quinolinone H), 15.26 (br s, 1H, COOH). Anal. calcd for C_30_H_22_FNO_5_: C, 72.72; H, 4.48; F, 3.83; N, 2.83%. Found: C, 72.65; H, 4.47; F, 3.82; N, 2.81%.

##### 1-(4-Fluorobenzyl)-4-oxo-6-(4-phenylbutoxy)-1,4-dihydroquinoline-3-carboxylic Acid (**4r**)

Compound **4r** was prepared from ethyl 1-(4-fluorobenzyl)-4-oxo-6-(4-phenylbutoxy)-1,4-dihydroquinoline-3-carboxylate by means of GP-D; 1 h; toluene; 60% as a white solid; 285 °C; IR ν 1624 (C=O), 1767 (C=O), 3059 (COOH) cm^–1^; ^1^H NMR (400 MHz DMSO-*d*_6_, δ) 1.65–1.69 (m, 2H, CH_2_), 2.52–2.57 (m, 2H, CH_2_), 4.06 (t, *J* = 7.0 Hz, 2H, CH_2_), 4.51 (t, *J* = 7.0 Hz, 2H, CH_2_), 5.76 (s, 2H, CH_2_), 7.09–7.26 (m, 9H, benzene H), 7.49 (d, *J* = 8.5 Hz, 1H, quinolinone H), 7.56 (d, *J* = 8.5 Hz, 1H, quinolinone H), 8.89 (s, 1H, quinolinone H), 9.12 (s, 1H, quinolinone H), 15.32 (br s, 1H, COOH). Anal. calcd for C_29_H_28_FNO_4_: C, 73.56; H, 5.96; F, 4.01; N, 2.96%. Found: C, 73.45; H, 5.95; F, 3.99; N, 2.97%.

##### 6-([1,1′-Biphenyl]-4-ylmethoxy)-1-(4-fluorobenzyl)-4-oxo-1,4-dihydroquinoline-3-carboxylic Acid (**4s**)

Compound **4s** was prepared from ethyl 6-([1,1′-biphenyl]-4-ylmethoxy)-1-(4-fluorobenzyl)-4-oxo-1,4-dihydroquinoline-3-carboxylate by means of GP-D; 2 h; toluene; 82% as a white solid; 275 °C; IR ν 1600 (C=O), 1765 (C=O), 3000 (COOH) cm^–1^; ^1^H NMR (400 MHz DMSO-*d*_6_, δ) 5.23 (s, 2H, CH_2_), 5.76 (s, 2H, CH_2_), 7.10–7.12 (m, 2H, benzene H), 7.23–7.30 (m, 2H, benzene H), 7.36–7.40 (m, 2H, benzene H), 7.47–7.51 (m, 3H, benzene H), 7.57–7.62 (m, 4H, benzene H, and quinolinone H), 7.76–7.78 (m, 3H, benzene H, and quinolinone H), 9.12 (s, 1H, quinolinone H), 15.18 (br s, 1H, OH). Anal. calcd for C_30_H_22_FNO_4_: C, 75.15; H, 4.62; F, 3.96; N, 2.92%. Found: C, 75.23; H, 4.64; F, 3.94; N, 2.92%.

##### 6-((4-(Benzyloxy)benzyl)oxy)-1-(4-fluorobenzyl)-4-oxo-1,4-dihydroquinoline-3-carboxylic Acid (**4t**)

Compound **4t** was prepared from ethyl 6-((4-(benzyloxy)benzyl)oxy)-1-(4-fluorobenzyl)-4-oxo-1,4-dihydroquinoline-3-carboxylate by means of GP-D; 2 h; toluene; 100% as a yellow solid; 230 °C; IR ν 1600 (C=O), 1807 (C=O), 3025 (COOH) cm^–1^; ^1^H NMR (400 MHz DMSO-*d*_6_, δ) 5.01 (s, 2H, CH_2_), 5.08 (s, 2H, CH_2_), 5.76 (s, 2H, CH_2_), 6.93–6.95 (m, 2H, benzene H), 7.07–7.11 (m, 3H, benzene H), 7.21–7.41 (m, 9H, benzene H, and quinolinone H), 7.51 (d, *J* = 8.5 Hz, 1H, quinolinone H), 7.73 (s, 1H, quinolinone H), 9.12 (s, 1H, quinolinone), 15.21 (br s, 1H, COOH). Anal. calcd for C_31_H_24_FNO_5_: C, 73.07; H, 4.75; F, 3.73; N, 2.75%. Found: C, 73.11; H, 4.74; F, 3.72; N, 2.74%.

##### Ethyl 1-(4-Fluorobenzyl)-4-oxo-1,4-dihydroquinoline-3-carboxylate (**5a**)

Synthesis, analytical, and spectroscopic data are reported in the literature.^41^

##### Ethyl 6-Cyano-1-(4-fluorobenzyl)-4-oxo-1,4-dihydroquinoline-3-carboxylate (**5b**)

Compound **5b** was prepared from ethyl 6-cyano-4-oxo-1,4-dihydroquinoline-3-carboxylate by means of GP-C; 2 h; ethyl acetate; ethanol; 93% as a yellow solid; 223 °C; IR ν 1592 (C=O), 1720 (C=O), 2231 (CN) cm^–1^; ^1^H NMR (400 MHz DMSO-*d*_6_, δ) 1.30 (t, *J* = 7.0 Hz, 3H, CH_3_), 4.26 (q, *J* = 7.0 Hz, 2H, CH_2_), 5.68 (s, 2H, CH_2_), 7.18–7.21 (m, 2H, benzene H), 7.32–7.36 (m, 2H, benzene H), 7.80 (d, *J* = 8.5 Hz, 1H, quinolinone H), 8.06 (d, *J* = 8.5 Hz, 1H, quinolinone H), 8.53 (s, 1H, quinolinone H), 8.95 (s, 1H, quinolinone H). Anal. calcd for C_20_H_15_FN_2_O_3_: C, 68.57; H, 4.32; F, 5.42; N, 8.00%. Found: C, 68.44; H, 4.31; F, 5.43; N, 8.01%.

##### Ethyl 1-(4-Fluorobenzyl)-4-oxo-6-(trifluoromethyl)-1,4-dihydroquinoline-3-carboxylate (**5c**)

Compound **5c** was prepared from ethyl 6-(methylsulfonyl)-4-oxo-1,4-dihydroquinoline-3-carboxylate by means of GP-C; 3 h; ethyl acetate; THF; 92% as a white solid; 211 °C; THF; IR ν 1612 (C=O) and 1706 (C=O) cm^–1^; ^1^H NMR (400 MHz DMSO-*d*_6_, δ) 1.31 (t, *J* = 7.0 Hz, 3H, CH_3_), 4.27 (q, *J* = 7.0 Hz, 2H, CH_2_), 5.77 (s, 2H, CH_2_), 7.13–7.18 (m, 2H, benzene H), 7.41–7.45 (m, 2H, benzene H), 7.86 (d, *J* = 8.5 Hz, 1H, quinolinone H), 7.94 (d, *J* = 8.5 Hz, 1H, quinolinone H), 8.61 (s, 1H, quinolinone H), 8.88 (s, 1H, quinolinone H). Anal. calcd for C_20_H_15_F_4_NO_3_: C, 61.07; H, 3.84; F, 19.32; N, 3.56%. Found: C, 61.30; H, 3.83; F, 19.36; N, 3.55%.

##### Ethyl 6-Acetyl-1-(4-fluorobenzyl)-4-oxo-1,4-dihydroquinoline-3-carboxylate (**5d**)

Synthesis, analytical, and spectroscopic data are reported in the literature.^38^

##### Ethyl 1-(4-Fluorobenzyl)-6-nitro-4-oxo-1,4-dihydroquinoline-3-carboxylate (**5e**)

Compound **5e** was prepared from ethyl 6-nitro-4-oxo-1,4-dihydroquinoline-3-carboxylate by means of GP-C; 2 h; ethyl acetate/methanol 95:5; ethanol; 86% as a brown solid; 211 °C; IR ν 1454 (NO_2_), 1617 (C=O), 1719 (C=O) cm^–1^; ^1^H NMR (400 MHz DMSO-*d*_6_, δ) 1.37 (t, *J* = 7.0 Hz, 3H, CH_3_), 4.29 (q, *J* = 7.0 Hz, 2H, CH_2_), 5.89 (s, 2H, CH_2_), 7.18–7.24 (m, 2H, benzene H), 7.34–7.39 (m, 2H, benzene H), 8.06 (d, *J* = 8.5 Hz, 1H, quinolinone H), 8.57 (d, *J* = 8.5 Hz, 1H, quinolinone H), 9.01 (s, 1H, quinolinone H), 9.35 (s, 1H, quinolinone H). Anal. calcd for C_19_H_15_FN_2_O_5_: C, 61.62; H, 4.08; F, 5.13; N, 7.56%. Found: C, 61.67; H, 4.09; F, 5.14; N, 7.55%.

##### Ethyl 1-(4-Fluorobenzyl)-6-(methylsulfonyl)-4-oxo-1,4-dihydroquinoline-3-carboxylate (**5f**)

Compound **5f** was prepared from ethyl 6-(methylsulfonyl)-4-oxo-1,4-dihydroquinoline-3-carboxylate by means of GP-C; 2 h; ethyl acetate; methanol; 88% as a brown solid; 224 °C; IR ν 1033 (SO_2_CH_3_), 1631 (C=O), 1733 (C=O) cm^–1^; ^1^H NMR (400 MHz DMSO_6_, δ) 1.29 (t, *J* = 7.0 Hz, 3H, CH_3_), 3.30 (s, 3H, CH_3_), 4.25 (q, *J* = 7.0 Hz, 2H, CH_2_), 5.70 (s, 2H, CH_2_), 7.17–7.21 (m, 2H, benzene H), 7.24–7.31 (m, 2H, benzene H), 7.87 (d, *J* = 8.5 Hz, 1H, quinolinone H), 8.13 (d, *J* = 7.0 Hz, 1H, quinolinone H), 8.68 (s, 1H, quinolinone H), 8.99 (s, 1H, quinolinone H). Anal. calcd for C_20_H_18_FNO_5_S: C, 59.55; H, 4.50; F, 4.71; N, 3.47; S, 7.95%. Found: C, 59.47; H, 4.49; F, 4.72; N, 3.48; S, 7.94%.

##### Ethyl 1-(4-Fluorobenzyl)-6-hydroxy-4-oxo-1,4-dihydroquinoline-3-carboxylate (**5g**)

Compound **5g** was prepared from ethyl 6-hydroxy-4-oxo-1,4-dihydroquinoline-3-carboxylate by means of GP-C; 2 h; chloroform/methanol 90:10; methanol; 70% as a brown solid; 220 °C; IR v 1554 (C=O), 1702 (C=O) cm^–1^; ^1^H NMR (400 MHz DMSO-*d*_6_, δ) 1.29 (t, *J* = 7.0 Hz, 3H, CH_3_), 4.25 (q, *J* = 7.0 Hz, 2H, CH_2_), 5.63 (s, 2H, CH_2_), 7.17–7.21 (m, 3H, benzene H, and quinolinone H), 7.28–7.31 (m, 2H, benzene H), 7.52 (d, *J* = 7.0 Hz, 1H, quinolinone H), 7.58 (s, 1H, quinolinone H), 8.83 (s, 1H, quinolinone H), 10.06 (br s, 1H, OH). Anal. calcd for C_17_H_12_FNO_4_: C, 65.18; H, 3.86; F, 6.06; N, 4.47%. Found: C, 65.27; H, 3.87; F, 6.07; N, 4.45%.

##### Ethyl 1-(4-Fluorobenzyl)-6-methoxy-4-oxo-1,4-dihydroquinoline-3-carboxylate (**5h**)

Compound **5h** was prepared from ethyl 6-methoxy-4-oxo-1,4-dihydroquinoline-3-carboxylate by means of GP-C; 2 h; chloroform/methanol 90:10; methanol; 82% as a brown solid; 225 °C; IR v 1557 (C=O) and 1705 (C=O) cm^–1^; ^1^H NMR (400 MHz DMSO-*d*_6_, δ) 1.30 (t, *J* = 7.0 Hz, 3H, CH_3_), 3.84 (s, 3H, CH_3_), 4.25 (q, *J* = 7.0 Hz, 2H, CH_2_), 5.66 (s, 2H, CH_2_), 7.17–7.19 (m, 2H, benzene H), 7.28–7.32 (m, 3H, benzene H, and quinolinone H), 7.60 (d, *J* = 8.5 Hz, 1H, quinolinone H), 7.66 (s, 1H, quinolinone H), 8.87 (s, 1H, quinolinone H). Anal. calcd for C_18_H_14_FNO_4_: C, 66.05; H, 4.31; F, 5.80; N, 4.28%. Found: C, 66.31; H, 4.30; F, 5.82; N, 4.27%.

##### Ethyl 1-(4-Fluorobenzyl)-4-oxo-6-phenoxy-1,4-dihydroquinoline-3-carboxylate (**5i**)

Compound **5i** was prepared from ethyl 4-oxo-6-phenoxy-1,4-dihydroquinoline-3-carboxylate by means of GP-C; 3 h; ethyl acetate/methanol 90:10; methanol; 73% as a yellow solid; >300 °C; IR ν 1556 (C=O) and 1703 (C=O) cm^–1^; ^1^H NMR (400 MHz DMSO-*d*_6_, δ) 1.27 (t, *J* = 7.0 Hz, 3H, CH_3_), 4.19–4.24 (q, *J* = 7.0 Hz, 2H, CH_2_), 5.66 (s, 2H, CH_2_), 7.09–7.11 (m, 2H, benzene), 7,17–7,24 (m, 3H, benzene H), 7.30–7.34 (m, 2H, benzene H), 7.14–7.47 (m, 2H, benzene H), 7.58–7.62 (m, 2H, quinolinone H), 7.71 (s, 1H, quinolinone H), 8.89 (s, 1H, quinolinone H). Anal. calcd for C_25_H_20_FNO_4_: C, 71.93; H, 4.83; F, 4.55; N, 3.36%. Found: C, 71.80; H, 4.84; F, 4.56; N, 3.37%.

##### Ethyl 6-(3-(Dimethylamino)propoxy)-1-(4-fluorobenzyl)-4-oxo-1,4-dihydroquinoline-3-carboxylate (**5j**)

Compound **5j** was prepared from ethyl 6-(3-(dimethylamino)propoxy)-4-oxo-1,4-dihydroquinoline-3-carboxylate by means of GP-C; 2 h; chloroform/methanol 90:10; methanol; 70% as a brown solid; >300°C; IR v 1554 (CO) and 1702 (CO) cm^–1^; ^1^H NMR (400 MHz DMSO-*d*_6_, δ) 1.35 (t, *J* = 7.0 Hz, 3H, CH_3_), 1.89–1.96 (m, 2H, CH_2_), 2.19 (s, 3H, CH_3_), 2.20 (s, 3H, CH_3_), 2.40 (t, *J* = 7.0 Hz, 2H, CH_2_), 4.13 (t, *J* = 7.0 Hz, 2H, CH_2_), 4.29 (q, *J* = 7.0 Hz, 2H, CH_2_), 5.71 (s, 2H, CH_2_), 7.22–7.27 (m, 2H, benzene H), 7.33–7.37 (m, 3H, benzene H, and quinolinone H), 7.65 (d, *J* = 8.5 Hz, 1H, quinolinone H), 7.70 (s, 1H, quinolinone H), 8.92 (s, 1H, quinolinone H). Anal. calcd for C_22_H_23_FN_2_O_4_: C, 66.32; H, 5.82; F, 4.77; N, 7.03%. Found: C, 66.45; H, 5.83; F, 4.76; N, 7.05%.

##### (*E*)-Ethyl 1-(4-Fluorobenzyl)-4-oxo-6-(3-oxo-3-phenylprop-1-en-1-yl)-1,4-dihydroquinoline-3-carboxylate (**5k**)

Compound **5k** was prepared from (*E*)-ethyl 4-oxo-6-(3-oxo-3-phenylprop-1-en-1-yl)-1,4-dihydroquinoline-3-carboxylate by means of GP-C; 2 h; ethyl acetate; acetonitrile; 80% as a yellow solid; 278 °C; IR ν 1570 (C=O), 1582 (C=O), 1684 (C=O) cm^–1^; ^1^H NMR (400 MHz DMSO-*d*_6_, δ) 1.28 (t, *J* = 7.0 Hz, 3H, CH_3_), 4.25 (q, *J* = 7.0 Hz, 2H, CH_2_), 5.69 (s, 2H, CH_2_), 7.17–7.21 (m, 2H, benzene H), 7.31–7.35 (m, 2H, benzene H), 7.54–7.57 (m, 2H, benzene H), 7.64–7.70 (t, *J* = 8.0 Hz, 1H, benzene H), 7.79–7.83 (m, 3H, benzene H, and alkene H), 7.98 (d, *J* = 16.0 Hz, 1H, alkene H), 8.15 (d, *J* = 8.5 Hz, 1H, quinolinone H), 8.25 (d, *J* = 7.0 Hz, 1H, quinolinone H), 8.55 (s, 1H, quinolinone), 8.91 (s, 1H, quinolinone). Anal. calcd for C_26_H_18_FNO_4_: C, 73.06; H, 4.24; F, 4.44; N, 3.28%. Found: C, 73.17; H, 4.25; F, 4.43; N, 3.27%.

##### Ethyl 6-(Benzyloxy)-1-(4-fluorobenzyl)-4-oxo-1,4-dihydroquinoline-3-carboxylate (**5l**)

Compound **5l** was prepared from ethyl 6-(benzyloxy)-4-oxo-1,4-dihydroquinoline-3-carboxylate by means of GP-C; 2 h; ethyl acetate; ethanol; 90% as a brown solid; >300 °C; IR ν 1595 (C=O), 1722 (C=O) cm^–1^; ^1^H NMR (400 MHz DMSO-*d*_6_, δ) 1.39 (t, *J* = 7.0 Hz, 3H, CH_3_), 4.37 (q, *J* = 7.0 Hz, 2H, CH_2_), 5.06 (s, 2H, CH_2_), 5.35 (s, 2H, CH_2_), 7.00–7.05 (m, 4H, benzene H), 7.10–7.14 (m, 2H, benzene H), 7.16–7.26 (m, 3H, benzene H), 7.39–7.45 (m, 2H, quinolinone H), 7.96 (s, 1H, quinolinone H), 8.52 (s, 1H, quinolinone H). Anal. calcd for C_26_H_22_FNO_4_: C, 72.38; H, 5.14; F, 4.40; N, 3.25%. Found: C, 72.56; H, 5.15; F, 4.41; N, 3.26%.

##### Ethyl 6-((2,3-Dichlorobenzyl)oxy)-1-(4-fluorobenzyl)-4-oxo-1,4-dihydroquinoline-3-carboxylate (**5m**)

Compound **5m** was prepared from ethyl 6-((2,3-dichlorobenzyl)oxy)-4-oxo-1,4-dihydroquinoline-3-carboxylate by means of GP-C; 2 h; ethyl acetate; cyclohexane; 65% as a brown solid; 267 °C; IR ν 1626 (C=O), 1738 (C=O) cm^–1^; ^1^H NMR (400 MHz DMSO-*d*_6_, δ) 1.30 (t, *J* = 7.0 Hz, 3H, CH_3_), 4.25 (q, *J* = 7.0 Hz, 2H, CH_2_), 5.30 (s, 2H, CH_2_), 5.67 (s, 2H, CH_2_), 7.17–7.22 (m, 2H, benzene H), 7.29–7.31 (m, 2H, benzene H), 7.40–7.44 (m, 2H, benzene H, and quinolinone H), 7.58–7.68 (m, 3H, benzene H, and quinolinone H), 7.75 (s, 1H, quinolone H), 8.88 (s, 1H, quinolinone H). Anal. calcd for C_26_H_20_Cl_2_FNO_4_: C, 62.41; H, 4.03; Cl, 14.17; F, 3.80; N, 2.80%. Found: C, 62.56; H, 4.04; Cl, 14.21; F, 3.79; N, 2.81%.

##### Ethyl 6-((3,4-Dichlorobenzyl)oxy)-1-(4-fluorobenzyl)-4-oxo-1,4-dihydroquinoline-3-carboxylate (**5n**)

Compound **5n** was prepared from ethyl 6-((3,4-dichlorobenzyl)oxy)-4-oxo-1,4-dihydroquinoline-3-carboxylate by means of GP-C; 3 h; ethyl acetate; benzene; 40% as a brown solid; 273 °C; IR ν 1615 (C=O), 1732 (C=O) cm^–1^; ^1^H NMR (400 MHz DMSO-*d*_6_, δ) 1.40 (t, *J* = 7.0 Hz, 3H, CH_3_), 4.40 (q, *J* = 7.0 Hz, 2H, CH_2_), 5.06 (s, 2H, CH_2_), 5.64 (s, 2H, CH_2_), 7.96–7.22 (m, 2H, benzene H), 7.29–7.31 (m, 2H, benzene H), 7.40–7.44 (m, 2H, benzene H, and quinolinone H), 7.58–7.68 (m, 3H, benzene H, and quinolinone H), 7.75 (s, 1H, quinolone H), 8.88 (s, 1H, quinolinone H). Anal. calcd for C_26_H_20_Cl_2_FNO_4_: C, 62.41; H, 4.03; Cl, 14.17; F, 3.97; N, 2.80%. Found: C, 62.56; H, 4.06; Cl, 14.20; F, 3.98; N, 2.81%.

##### Ethyl 1-(4-Fluorobenzyl)-6-(naphthalen-1-ylmethoxy)-4-oxo-1,4-dihydroquinoline-3-carboxylate (**5o**)

Compound **5o** was prepared from ethyl 6-(naphthalen-1-ylmethoxy)-4-oxo-1,4-dihydroquinoline-3-carboxylate by means of GP-C; 2 h; ethyl acetate; toluene; 59% as a yellow solid; 222 °C; IR ν 1598 (C=O), 1730 (C=O) cm^–1^; ^1^H NMR (400 MHz DMSO-*d*_6_, δ) 1.21 (t, *J* = 7.0 Hz, 3H, CH_3_), 4.15 (q, *J* = 7.0 Hz, 2H, CH_2_), 5.21 (s, 2H, CH_2_), 5.57 (s, 2H, CH_2_), 7.08–7.13 (m, 3H, benzene H, and naphthalene H), 7.20–7.24 (m, 2H, naphthalene H), 7.29–7.35 (m, 2H, quinolinone H), 7.41–7.60 (m, 3H, benzene H, and naphthalene H), 7.79 (d, *J* = 8.5 Hz, 1H, naphthalene H), 7.85–7.91 (m, 2H, naphthalene H), 8.01 (s, 1H, quinolinone H), 8.89 (s, 1H, quinolinone). Anal. calcd for C_30_H_24_FNO_4_: C, 74.83; H, 5.02; F, 3.95; N, 2.91%. Found: C, 74.90; H, 5.00; F, 3.96; N, 2.90%.

##### Ethyl 1-(4-Fluorobenzyl)-6-(naphthalen-2-ylmethoxy)-4-oxo-1,4-dihydroquinoline-3-carboxylate (**5p**)

Compound **5p** was prepared from ethyl 6-(naphthalen-2-ylmethoxy)-4-oxo-1,4-dihydroquinoline-3-carboxylate by means of GP-C; 3 h; ethyl acetate; toluene; 59% as a brown solid; 284 °C; IR ν 1630 (C=O), 1716 (C=O) cm^–1^; ^1^H NMR (400 MHz DMSO-*d*_6_, δ) 1.35 (t, *J* = 7.0 Hz, 3H, CH_3_), 4.28 (q, *J* = 7.0 Hz, 2H, CH_2_), 5.43 (s, 2H, CH_2_), 5.71 (s, 2H, CH_2_), 7.22–7.28 (m, 2H, benzene H), 7.33–7.36 (m, 2H, benzene H), 7.45 (d, *J* = 8.5 Hz, 1H, quinolinone H), 7.57–7.59 (m, 2H, naphthalene H), 7.64–7.69 (m, 2H, naphthalene H), 7.81 (s, 1H, quinolinone H), 7.96–8.05 (m, 4H, naphthalene H), 8.92 (s, 1H, quinolinone H). Anal. calcd for C_30_H_24_FNO_4_: C, 74.83; H, 5.02; F, 3.95; N, 2.91%. Found: C, 74.99; H, 5.03; F, 3.96; N, 2.90%.

##### Ethyl 6-(Benzo[*d*][1,3]dioxol-5-ylmethoxy)-1-(4-fluorobenzyl)-4-oxo-1,4-dihydroquinoline-3-carboxylate (**5q**)

Compound **5q** was prepared from ethyl 6-(benzo[*d*][1,3]dioxol-5-ylmethoxy)-4-oxo-1,4-dihydroquinoline-3-carboxylate by means of GP-C; 3 h; ethyl acetate; 2-propanol; 71% as a brown solid; 291 °C; IR ν 1608 (C=O), 1718 (C=O) cm^–1^; ^1^H NMR (400 MHz DMSO-*d*_6_, δ) 1.26 (t, *J* = 7.0 Hz, 3H, CH_3_), 4.25 (q, *J* = 7.0 Hz, 2H, CH_2_), 5.09 (s, 2H, CH_2_), 5.66 (s, 2H, CH_2_), 6.02 (s, 2H, CH_2_), 6.88–6.97 (m, 2H, benzodioxolane H), 7.03 (s, 1H, benzodioxolane H), 7.17–7.22 (m, 2H, benzene H), 7.27–7.30 (m, 2H, benzene H), 7.37 (d, *J* = 8.5 Hz, 1H, quinolinone H), 7.60 (d, 1H, *J* = 8.5 Hz, quinolone H), 7.75 (s, 1H, quinolone H), 8.86 (s, 1H, quinolinone H). Anal. calcd for C_27_H_22_FNO_6_: C, 68.21; H, 4.66; F, 4.00; N, 2.95%. Found: C, 68.34; H, 4.67; F, 4.01; N, 2.96%.

##### Ethyl 1-(4-Fluorobenzyl)-4-oxo-6-(4-phenylbutoxy)-1,4-dihydroquinoline-3-carboxylate (**5r**)

Compound **5r** was prepared from ethyl 4-oxo-6-(4-phenylbutoxy)-1,4-dihydroquinoline-3-carboxylate by means of GP-C; 3 h; ethyl acetate; toluene; 90% as a brown solid; 285 °C; IR ν 1606 (C=O), 1725 (C=O) cm^–1^; ^1^H NMR (400 MHz DMSO-*d*_6_, δ) 1.19 (q, *J* = 7.0 Hz, 3H, CH_3_), 1.65–1.68 (m, 4H, CH_2_), 2.52–2.57 (m, 2H, CH_2_), 3.99 (t, *J* = 7.0 Hz, 2H, CH_2_), 4.15 (t, *J* = 7.0 Hz, 2H, CH_2_), 5.56 (s, 2H, CH_2_), 7.06–7.29 (m, 9H, benzene H), 7.49 (d, *J* = 8.5 Hz, 1H, quinolinone H), 7.57 (s, 1H, quinolinone H), 7.63–7.65 (d, 1H, quinolinone H), 8.76 (s, 1H, quinolinone H). Anal. calcd for C_29_H_28_FNO_4_: C, 73.56; H, 5.96; F, 4.01; N, 2.96%. Found: C, 73.67; H, 5.95; F, 4.00; N, 2.95%.

##### Ethyl 6-([1,1′-Biphenyl]-4-ylmethoxy)-1-(4-fluorobenzyl)-4-oxo-1,4-dihydroquinoline-3-carboxylate (**5s**)

Compound **5s** was prepared from ethyl 6-([1,1′-biphenyl]-4-ylmethoxy)-4-oxo-1,4-dihydroquinoline-3-carboxylate by means of GP-C; 2 h; ethyl acetate; ethanol; 88% as a brown solid; 285 °C; IR ν 1591 (C=O), 1748 (C=O) cm^–1^; ^1^H NMR (400 MHz DMSO-*d*_6_, δ) 1.21 (t, *J* = 7.0 Hz, 3H, CH_3_), 4.15 (q, *J* = 7.0 Hz, 2H, CH_2_), 5.17 (s, 2H, CH_2_), 5.57 (s, 2H, CH_2_), 7.08–7.12 (m, 2H, benzene H), 7.19–7.23 (m, 2H, benzene H), 7.28–7.33 (m, 2H, benzene H), 7.36–7.61 (m, 9H, benzene H, and quinilinone H), 7.69 (s, 1H, quinolinone H), 8.77 (s, 1H, quinolinone). Anal. calcd for C_32_H_26_FNO_4_: C, 75.73; H, 5.16; F, 3.74; N, 2.76%. Found: C, 75.65; H, 5.15; F, 3.75; N, 2.75%.

##### Ethyl 6-((4-(Benzyloxy)benzyl)oxy)-1-(4-fluorobenzyl)-4-oxo-1,4-dihydroquinoline-3-carboxylate (**5t**)

Compound **5t** was prepared from ethyl 6-((4-(benzyloxy)benzyl)oxy)-4-oxo-1,4-dihydroquinoline-3-carboxylate by means of GP-C; 2.5 h; ethyl acetate; toluene; 69% as a yellow solid; 256 °C; IR ν 1600 (C=O), 1730 (C=O) cm^–1^; ^1^H NMR (400 MHz DMSO-*d*_6_, δ) 1.21 (t, *J* = 7.0 Hz, 3H, CH_3_), 4.15 (q, *J* = 7.0 Hz, 2H, CH_2_), 5.01 (s, 2H, CH_2_), 5.06 (s, 2H, CH_2_), 5.57 (s, 2H, CH_2_), 6.93 (d, *J* = 8.0 Hz, 2H, benzene H), 7.09 (t, *J* = 8.0 Hz, 1H, benzene H), 7.21–7.41 (m, 11H, benzene H, and quinolinone H), 7.50 (d, *J* = 8.5 Hz, 1H, quinolinone H), 7.66 (s, 1H, quinolinone H), 8.78 (s, 1H, quinolinone H). Anal. calcd for C_33_H_28_FNO_5_: C, 73.73; H, 5.25; F, 3.53; N, 2.61%. Found: C, 73.65; H, 5.26; F, 3.52; N, 2.60%.

##### (*E*)-3-(4-Aminophenyl)-1-phenylprop-2-en-1-one (**6**)

Synthesis, analytical, and spectroscopic data are reported in the literature.^37^

##### Synthesis of (*E*)-Diethyl 2-(((4-(3-oxo-3-phenylprop-1-en-1-yl)phenyl)amino)methylene)malonate (**7**)

(*E*)-3-(4-Aminophenyl)-1-phenylprop-2-en-1-one (2.20 mmol) was suspended in diethyl ethoxymethylenemalonate (0.44 mL; 2.20 mmol), and the reaction was stirred at 90 °C for 3 h and monitored by TLC. Upon completion of the reaction, the mixture was cooled down and poured into *n*-hexane (20 mL). The resulting precipitate was filtered, washed three times with *n*-hexane (5 mL) and petroleum ether, and dried under an IR lamp to afford the pure product as a brown solid (90%). Chromatography eluent: *n*-hexane/ethyl acetate 60:40. Recrystallization solvent: cyclohexane; mp 150 °C; IR ν 1589 (C=O), 1609 (C=O), 1681 (C=O), 2984 (NH) cm^–1^; ^1^H NMR (400 MHz, CDCl_3_, δ) 1.36–1.40 (m, 6H, CH_3_), 4.24–4.35 (m, 4H, CH_2_), 7.18 (d, *J* = 8.0 Hz, 2H, benzene H), 7.50 (d, *J* = 16.0 Hz, 1H, alkene H), 7.55 (d, *J* = 8.0 Hz, 2H, benzene H), 7.59 (d, *J* = 16.0 Hz, 1H, alkene H), 7.67 (d, *J* = 8.0 Hz, 2H, benzene H), 7.79 (t, *J* = 8.0 Hz, 1H, benzene H), 8.02 (d, *J* = 8.0 Hz, 2H, benzene H), 8,55 (d, *J* = 7.0 Hz, 1H, alkene H) 11.11 (d, *J* = 7.0 Hz, 1H, NH). Anal. calcd for C_23_H_23_NO_5_: C, 70.21; H, 5.89; N, 3.56%. Found: C, 70.25; H, 5.90; N, 3.55%.

##### Ethyl 4-Oxo-1,4-dihydroquinoline-3-carboxylate (**8a**)

Synthesis, analytical, and spectroscopic data are reported in the literature.^41^

##### Ethyl 6-Cyano-4-oxo-1,4-dihydroquinoline-3-carboxylate (**8b**)

Synthesis, analytical, and spectroscopic data are reported in the literature.^43^

##### Ethyl 6-(Trifluromethyl)-4-oxo-1,4-dihydroquinoline-3-carboxylate (**8c**)

Synthesis, analytical, and spectroscopic data are reported in the literature.^44^

##### Ethyl 6-Acetyl-4-oxo-1,4-dihydroquinoline-3-carboxylate (**8d**)

Synthesis, analytical, and spectroscopic data are reported in the literature.^38^

##### Ethyl 6-Nitro-4-oxo-1,4-dihydroquinoline-3-carboxylate (**8e**)

Synthesis, analytical, and spectroscopic data are reported in the literature.^42^

##### Ethyl 6-(Methylsulfonyl)-4-oxo-1,4-dihydroquinoline-3-carboxylate (**8f**)

Synthesis, analytical, and spectroscopic data are reported in the literature.^47^

##### Ethyl 6-Hydroxy-4-oxo-1,4-dihydroquinoline-3-carboxylate (**8g**)

Synthesis, analytical, and spectroscopic data are reported in the literature.^45^

##### Ethyl 6-Methoxy-4-oxo-1,4-dihydroquinoline-3-carboxylate (**8h**)

Synthesis, analytical, and spectroscopic data are reported in the literature.^46^

##### Ethyl 4-Oxo-6-phenoxy-1,4-dihydroquinoline-3-carboxylate (**8i**)

Synthesis, analytical, and spectroscopic data are reported in the literature.^48^

##### Ethyl 6-(3-(Dimethylamino)propoxy)-4-oxo-1,4-dihydroquinoline-3-carboxylate (**8j**)

Compound **8j** was prepared from (*E*)-diethyl diethyl 2-(((4(4(dimethylamino)butoxy)phenyl)amino)methylene)malonate by means of GP-B; 3 h; ethanol; 90% as a yellow solid; 293 °C; IR ν 1623 (C=O), 1717 (C=O), 2965 (NH) cm^–1^; ^1^H NMR (400 MHz DMSO-*d*_6_, δ) 1.36 (t, *J* = 7.0 Hz, 3H, CH_3_), 1.89–1.96 (m, 2H, CH_2_), 2.15 (s, 3H, CH_3_), 2.16 (s, 3H, CH_3_), 2.42 (t, *J* = 7.0 Hz, 2H, CH_2_), 4.15 (t, *J* = 7.0 Hz, 2H, CH_2_), 4.32 (q, *J* = 7.0 Hz, 2H, CH_2_), 7.39 (d, *J* = 8.5 Hz, 1H, quinolinone H), 7.65 (d, *J* = 8.5 Hz, 1H, quinolinone H), 7.71 (s, 1H, quinolinone H), 8.92 (s, 1H, quinolinone H), 10.31 (br s, 1H, NH). Anal. calcd for C_17_H_22_N_2_O_4_: C, 64.13; H, 6.97; N, 8.80%. Found: C, 64.24; H, 6.95; N, 8.81%.

##### (*E*)-Ethyl 4-Oxo-6-(3-oxo-3-phenylprop-1-en-1-yl)-1,4-dihydroquinoline-3-carboxylate (**8k**)

Compound **8k** was prepared from (*E*)-diethyl 2-(((4-(3-oxo-3-phenylprop-1-en-1-yl)phenyl)amino)methylene)malonate by means of GP-B; 2 h; ethanol; 100% as a yellow solid; 289 °C; IR ν 1604 (C=O), 1661 (C=O), 1708 (C=O), 2975 (NH) cm^–1^; ^1^H NMR (400 MHz DMSO-*d*_6_, δ) 1.28 (t, *J* = 7.0 Hz, 3H, CH_3_), 4.23 (q, *J* = 7.0 Hz, 2H, CH_2_), 7.19 (d, *J* = 8.0 Hz, 2H, benzene H), 7.38–7.45 (m, 4H, benzene H, and alkene H), 7.50 (d, *J* = 16.0 Hz, 1H, alkene H), 7.67 (d, *J* = 8.5 Hz, 1H, quinolinone H), 7.79 (d, *J* = 8.5 Hz, 1H, quinolinone H), 8.01 (s, 1H, quinolinone H), 8.53 (s, 1H, quinolinone H), 11.19 (br s, 1H, NH). Anal. calcd for C_21_H_17_NO_4_: C, 72.61; H, 4.93; N, 4.03%. Found: C, 72.67; H, 4.92; N, 4.04%.

##### Ethyl 6-(Benzyloxy)-4-oxo-1,4-dihydroquinoline-3-carboxylate (**8l**)

Synthesis, analytical, and spectroscopic data are reported in the literature.^49^

##### Ethyl 6-((2,3-Dichlorobenzyl)oxy)-4-oxo-1,4-dihydroquinoline-3-carboxylate (**8m**)

Compound **8m** was prepared from diethyl 2-(((4-((2,3-dichlorobenzyl)oxy)phenyl)amino)methylene)malonate by means of GP-B; 2 h; toluene; 92% as a brown solid; 198 °C; IR ν 1609 (C=O), 1714 (C=O), 2978 (NH) cm^–1^; ^1^H NMR (400 MHz DMSO-*d*_6_) δ 1.30 (t, *J* = 7.0 Hz, 3H, CH_3_), 4.22 (t, 2H, *J* = 7.0 Hz, CH_2_) 5.32 (s, 2H, CH_2_), 7.43–7.47 (m, 2H, benzene H, and quinolinone H), 7.60–7.65 (m, 3H, benzene H, and quinolinone H), 7.72 (s, 1H, quinolone H), 8.88 (s, 1H, quinolinone H), 12.34 (br s, 1H, NH). Anal. calcd for C_19_H_15_Cl_2_NO_4_: C, 58.18; H, 3.85; Cl, 18.08; N, 3.57%. Found: C, 58.20; H, 3.84; Cl, 18.06; N, 3.56%.

##### Ethyl 6-((3,4-Dichlorobenzyl)oxy)-4-oxo-1,4-dihydroquinoline-3-carboxylate (**8n**)

Compound **8n** was prepared from diethyl 2-(((4-((3,4-dichlorobenzyl)oxy)phenyl)amino)methylene)malonate by means of GP-B; 3 h; toluene; 96% as a brown solid; 210 °C; IR ν 1608 (C=O), 1714 (C=O), 2989 (NH) cm^–1^; ^1^H NMR (400 MHz DMSO-*d*_6_, δ) 1.39 (t, *J* = 7.0 Hz, 3H, CH_3_), 4.46 (t, *J* = 7.0 Hz, 2H, CH_2_) 5.27 (s, 2H, CH_2_), 7.43–7.47 (m, 2H, benzene H, and quinolinone H), 7.60–7.65 (m, 3H, benzene H, and quinolinone H), 7.76 (s, 1H, quinolone H), 8.71 (s, 1H, quinolinone H), 9.22 (br s, 1H, NH). Anal. calcd for C_19_H_15_Cl_2_NO_4_: C, 58.18; H, 3.85; Cl, 18.08; N, 3.57%. Found: C, 58.32; H, 3.86; Cl, 18.11; N, 3.58%.

##### Ethyl 6-(Naphthalen-1-ylmethoxy)-4-oxo-1,4-dihydroquinoline-3-carboxylate (**8o**)

Compound **8o** was prepared from diethyl 2-(((4-(naphthalen-1-ylmethoxy)phenyl)amino)methylene)malonate by means of GP-B; 2 h; ethanol; 100% as a yellow solid; 191 °C; IR ν 1620 (C=O), 1730 (C=O), 2926 (NH) cm^–1^; ^1^H NMR (400 MHz DMSO-*d*_6_, δ) 1.17 (t, *J* = 7.0 Hz, 3H, CH_3_), 4.13 (q, *J* = 7.0 Hz, 2H, CH_2_), 5.58 (s, 2H, CH_2_), 6.91–6.93 (m, 2H, naphthalene H), 7.03–7.07 (m, 2H, naphthalene H), 7.41–7.60 (m, 3H, naphthalene H), 7.84–8.02 (m, 3H, quinolinone H), 8.42 (s, 1H, quinolinone H), 12.23 (br s, 1H, NH). Anal. calcd for C_23_H_19_NO_4_: C, 73.98; H, 5.13; N, 3.75%. Found: C, 74.10; H, 5.14; N, 3.74%.

##### Ethyl 6-(Naphthalen-2-ylmethoxy)-4-oxo-1,4-dihydroquinoline-3-carboxylate (**8p**)

Compound **8p** was prepared from diethyl 2-(((4-(naphthalen-2-ylmethoxy)phenyl)amino)methylene)malonate by means of GP-B; 2 h; benzene; 95% as a brown solid; 222 °C; IR ν 1613 (C=O), 1720 (C=O), 2901 (NH) cm^–1^; ^1^H NMR (400 MHz DMSO-*d*_6_, δ) 1.30 (t, *J* = 7.0 Hz, 3H, CH_3_), 4.22 (t, *J* = 7.0 Hz, 2H, CH_2_) 5.39 (s, 2H, CH_2_), 7.60–7.63 (m, 2H, naphthalene H), 7.69–7.74 (m, 2H, naphthalene H), 7.77–8.05 (m, 6H, naphthalene H, and quinolinone H), 8.49 (s, 1H, quinolinone H), 12.30 (br s, 1H, NH). Anal. calcd for C_23_H_19_NO_4_: C, 73.98; H, 5.13; N, 3.75%. Found: C, 74.02; H, 5.14; N, 3.76%.

##### Ethyl 6-(Benzo[*d*][1,3]dioxol-5-ylmethoxy)-4-oxo-1,4-dihydroquinoline-3-carboxylate (**8q**)

Compound **8q** was prepared from diethyl 2-(((4-(benzo[*d*][1,3]dioxol-5-ylmethoxy)phenyl)amino)methylene)malonate by means of GP-B; 2 h; 2-propanol; 100% as a brown solid; 188 °C; IR ν 1622 (C=O), 1721 (C=O), 2954 (NH) cm^–1^; ^1^H NMR (400 MHz DMSO-*d*_6_, δ) 1.29 (t, *J* = 7.0 Hz, 3H, CH_3_), 4.25 (q, *J* = 7.0 Hz, 2H, CH_2_), 5.09 (s, 2H, CH_2_), 5.66 (s, 2H, CH_2_), 6.02 (s, 2H, CH_2_), 6.88–6.97 (m, 2H, benzodioxolane H), 7.06 (s, 1H, benzodioxolane H), 7.20–7.25 (m, 2H, benzene H), 7.30–7.33 (m, 2H, benzene H), 7.39 (d, *J* = 8.5 Hz, 1H, quinolinone H), 7.60–7.62 (d, *J* = 8.5 Hz, 1H, quinolone H), 7.75 (s, 1H, quinolone H), 8.86 (s, 1H, quinolinone H), 12.30 (br s, 1H, NH). Anal. calcd for C_20_H_17_NO_6_: C, 65.39; H, 4.66; N, 3.81; O, 26.13%. Found: C, 65.43; H, 4.67; N, 3.82%.

##### Ethyl 4-Oxo-6-(4-phenylbutoxy)-1,4-dihydroquinoline-3-carboxylate (**8r**)

Compound **8r** was prepared from diethyl 2-(((4-(4-phenylbutoxy)phenyl)amino)methylene)malonate by means of GP-B; 3 h; benzene; 91% as a brown solid; 200 °C; IR ν 1614 (C=O), 1725 (C=O), 2965 (NH) cm^–1^; ^1^H NMR (400 MHz DMSO-*d*_6_, δ) 1.20 (q, *J* = 7.0 Hz, 3H, CH_3_), 1.59–1.63 (m, 2H, CH_2_), 1.74–1.78 (m, 2H, CH_2_) 2.66 (t, *J* = 7.0 Hz, 2H, CH_2_), 4.04 (t, *J* = 7.0 Hz, 2H, CH_2_), 4.24 (q, *J* = 7.0 Hz, 2H, CH_2_), 7.19–7.24 (m, 5H, benzene H), 7.47 (d, *J* = 8.5 Hz, 1H, quinolinone H), 7.51 (s, 1H, quinolinone H), 7.60 (d, *J* = 8.5 Hz, 1H, quinolinone H), 8.89 (s, 1H, quinolinone H), 12.01 (br s, 1H, NH). Anal. calcd for C_22_H_23_NO_4_: C, 72.31; H, 6.34; N, 3.83%. Found: C, 72.40; H, 6.35; N, 3.84%.

##### Ethyl 6-([1,1′-Biphenyl]-4-ylmethoxy)-4-oxo-1,4-dihydroquinoline-3-carboxylate (**8s**)

Compound **8s** was prepared from diethyl 2-(((4-([1,1′-biphenyl]-4-ylmethoxy)phenyl)amino)methylene)malonate by means of GP-B; 2 h; benzene; 95% as a brown solid; 280 °C; IR ν 1604 (C=O), 1710 (C=O), 2900 (NH) cm^–1^; ^1^H NMR (400 MHz DMSO-*d*_6_, δ) 1.19 (t, *J* = 7.0 Hz, 3H, CH_3_), 4.13 (q, *J* = 7.0 Hz, 2H, CH_2_), 5.15 (s, 2H, CH_2_), 7.28–7.60 (m, 12H, benzene H), 8.22 (s, 1H, quinolinone H), 11.62 (br s, 1H, NH). Anal. calcd for C_25_H_21_NO_4_: C, 75.17; H, 5.30; N, 3.51%. Found: C, 75.34; H, 5.31; N, 3.50%.

##### Ethyl 6-((4-(Benzyloxy)benzyl)oxy)-4-oxo-1,4-dihydroquinoline-3-carboxylate (**8t**)

Compound **8t** was prepared from diethyl 2-(((4-((4-(benzyloxy)benzyl)oxy)phenyl)amino)methylene)malonate by means of GP-B; 3 h; toluene; 94% as a brown solid; 198 °C; IR ν 1609 (C=O), 1718 (C=O), 2926 (NH) cm^–1^; ^1^H NMR (400 MHz DMSO-*d*_6_, δ) 1.19 (t, *J* = 7.0 Hz, 3H, CH_3_), 4.12 (q, *J* = 7.0 Hz, 2H, CH_2_), 5.03 (s, 2H, CH_2_), 5.10 (s, 2H, CH_2_); 6.91–6.95 (m, 2H, benzene H), 7.24–7.37 (m, 7H, benzene H), 7.48–7.50 (m, 2H, quinolinone H), 7.56 (s, 1H, quinolinone H), 8.40 (s, 1H, quinolinone H), 12.20 (br s, 1H, NH). Anal. calcd for C_26_H_23_NO_5_: C, 72.71; H, 5.40; N, 3.26%. Found: C, 72.87; H, 5.41; N, 3.25%.

##### Diethyl 2-(((4-Hydroxyphenyl)amino)methylene)malonate (**9**)

Synthesis, analytical, and spectroscopic data are reported in the literature.^50^

##### Diethyl 2-(((4-(3-(Dimethylamino)propoxy)phenyl)amino)methylene)malonate (**10a**)

Compound **10a** was prepared from 2-(((4-hydroxyphenyl)amino)methylene)malonate by means of GP-A; 3-dimethylamino-1-propyl chloride; 2 h; ethyl acetate; ethanol; 62% as a brown solid; 134 °C; IR ν 1718 (C=O), 2926 (NH) cm^–1^; ^1^H NMR (400 MHz DMSO-*d*_6_, δ) 1.35–1.40 (m, 6H, CH_3_), 1.90–1.96 (m, 2H, CH_2_), 2.17 (s, 3H, CH_3_), 2.18 (s, 3H, CH_3_), 2.45 (t, *J* = 7.0 Hz, 2H, CH_2_), 4.17 (t, *J* = 7.0 Hz, 2H, CH_2_), 4.29–4.34 (m, 4H, CH_2_), 6.94 (d, *J* = 8.0 Hz, 2H, benzene H), 7.29 (d, *J* = 8.0 Hz, 2H, benzene H), 8.33 (s, 1H, alkene H), 10.70 (s, 1H, NH). Anal. calcd for C_19_H_28_N_2_O_5_: C, 62.62; H, 7.74; N, 7.69%. Found: C, 62.54; H, 7.73; N, 7.68%.

##### Diethyl 2-(((4-(Benzyloxy)phenyl)amino)methylene)malonate (**10b**)

Synthesis, analytical, and spectroscopic data are reported in the literature.^9^

##### Diethyl 2-(((4-((2,3-Dichlorobenzyl)oxy)phenyl)amino)methylene)malonate (**10c**)

Compound **10c** was prepared from 2-(((4-hydroxyphenyl)amino)methylene)malonate by means of GP-A; 2,3-dichlorobenzyl chloride; 2 h; *n*-hexane/ethyl acetate 70:30; ethanol; 67% as a white solid; 185 °C; IR ν 1718 (C=O), 2939 (NH) cm^–1^; ^1^H NMR (400 MHz DMSO-*d*_6_, δ) 1.23–1.32 (m, 6H, CH_3_), 4.14–4.26 (m, 4H, CH_2_), 5.09 (s, 2H, CH_2_), 6.91 (d, *J* = 8.0 Hz, 2H, benzene H), 7.02 (d, *J* = 8.0 Hz, 2H, benzene H), 7.8 (d, *J* = 8.0 Hz, 1H, benzene H), 7.36–7.40 (m, 2H, benzene H), 8.37 (s, 1H, alkene H), 10.92 (s, 1H, NH). Anal. calcd for C_21_H_21_Cl_2_NO_5_: C, 57.55; H, 4.83; Cl, 16.18; N, 3.20%. Found: C, 57.64; H, 4.82; Cl, 16.20; N, 3.21%.

##### Diethyl 2-(((4-((3,4-Dichlorobenzyl)oxy)phenyl)amino)methylene)malonate (**10d**)

Compound **10d** was prepared from 2-(((4-hydroxyphenyl)amino)methylene)malonate by means of GP-A; 3,4-dichlorobenzyl chloride; 3 h; *n*-hexane/ethyl acetate 70:30; ethanol; 70% as a white solid; 177 °C; IR ν 1715 (C=O), 2939 (NH) cm^–1^; ^1^H NMR (400 MHz DMSO-*d*_6_, δ) 1.23–1.26 (m, 6H, CH_3_), 4.10–4.20 (m, 4H, CH_2_), 5.12 (s, 2H, CH_2_), 7.04 (d, *J* = 8.0 Hz, 2H, benzene H), 7.34 (d, *J* = 8.0 Hz, 2H, benzene H), 7.44 (d, *J* = 8.0 Hz, 1H, benzene H), 7.66 (d, *J* = 8.0 Hz, 1H, benzene H), 7.71 (s, 1H, benzene H), 8.32 (s, 1H, alkene H), 10.71 (s, 1H, NH). Anal. calcd for C_21_H_21_Cl_2_NO_5_: C, 57.55; H, 4.83; Cl, 16.18; N, 3.20%. Found: C, 57.62; H, 4.82; Cl, 16.19; N, 3.21%.

##### Diethyl 2-(((4-(Naphthalen-1-ylmethoxy)phenyl)amino)methylene)malonate (**10e**)

Compound **10e** was prepared from 2-(((4-hydroxyphenyl)amino)methylene)malonate by means of GP-A; 1-(chloromethyl)naphthalene; 3 h; *n*-hexane/ethyl acetate 70:30; ethanol; 60% as a green solid; 124 °C; IR ν 1718 (C=O), 2926 (NH) cm^–1^; ^1^H NMR (400 MHz DMSO-*d*_6_, δ) 1.12–1.18 (m, 6H, CH_3_), 3.99–4.13 (m, 4H, CH_2_), 5.46 (s, 2H, CH_2_), 7.04 (d, *J* = 8.0 Hz, 2H, benzene H), 7.26 (d, *J* = 8.0 Hz, 2H, benzene H), 7.41–7.60 (m, 4H, naphthalene H), 7.84–8.02 (m, 3H, naphthalene H), 8.24 (d, *J* = 12.0 Hz, 1H, alkene), 10.63 (d, *J* = 12.0 Hz, 1H, NH). Anal. calcd for C_25_H_25_NO_5_: C, 71.58; H, 6.01; N, 3.34%. Found: C, 71.66; H, 6.02; N, 3.35%.

##### Diethyl 2-(((4-(Naphthalen-2-ylmethoxy)phenyl)amino)methylene)malonate (**10f**)

Compound **10f** was prepared from 2-(((4-hydroxyphenyl)amino)methylene)malonate by means of GP-A; 2-(chloromethyl)naphthalene; 3 h; *n*-hexane/ethyl acetate 70:30; ethanol; 80% as a white solid; 179 °C; IR ν 1712 (C=O), 2934 (NH) cm^–1^; ^1^H NMR (400 MHz DMSO-*d*_6_, δ) 1.21–1.27 (m, 6H, CH_3_), 4.10 (q, *J* = 7.0 Hz, 2H, CH_2_), 4.19 (q, *J* = 7.0 Hz, 2H, CH_2_), 5.28 (s, 2H, CH_2_), 7.09 (d, *J* = 8.0 Hz, 2H, benzene H), 7.33 (d, *J* = 8.0 Hz, 2H, benzene H), 7.52–7.59 (m, 3H, naphthalene H), 7.92–7.98 (m, 4H, naphthalene H), 8.35 (s, 1H, alkene H), 10.71 (s, 1H, NH). Anal. calcd for C_25_H_25_NO_5_: C, 71.58; H, 6.01; N, 3.34%. Found: C, 71.60; H, 6.02; N, 3.35%.

##### Diethyl 2-(((4-(Benzo[*d*][1,3]dioxol-5-ylmethoxy)phenyl)amino)methylene)malonate (**10g**)

Compound **10g** was prepared from 2-(((4-hydroxyphenyl)amino)methylene)malonate by means of GP-A; 5-(bromomethyl)-1,3-benzodioxole; 2.5 h; *n*-hexane/ethyl acetate 70:30; ethanol; 75% as a white solid; 182 °C; IR ν 1745 (C=O), 2909 (NH) cm^–1^; ^1^H NMR (400 MHz DMSO-*d*_6_, δ) 1.22–1.28 (m, 6H, CH_3_), 4.13 (q, *J* = 7.0 Hz, 2H, CH_2_), 4.19, (q, *J* = 7.0 Hz, 2H, CH_2_), 4.99 (s, 2H, CH_2_), 6.02 (s, 2H, CH_2_), 6.77–6.90 (m, 2H, benzodioxolane H), 6.93–6.97 (m, 3H, benzodioxolane H, and benzene H), 7.31 (d, *J* = 8.0 Hz, 2H, benzene H), 8.32 (d, *J* = 12.0 Hz, 1H, alkene H), 10.70 (d, *J* = 12.0 Hz, 1H, NH). Anal. calcd for C_22_H_23_NO_7_: C, 63.92; H, 5.61; N, 3.39%. Found: C, 64.09; H, 5.60; N, 3.40%.

##### Diethyl 2-(((4-(4-Phenylbutoxy)phenyl)amino)methylene)malonate (**10h**)

Compound **10h** was prepared from 2-(((4-hydroxyphenyl)amino)methylene)malonate by means of GP-A; 1-bromo-4-phenylbutane; 2 h; *n*-hexane/ethyl acetate 70:30; ethanol; 90% as a white solid; 165 °C; IR ν 1705 (C=O), 2934 (NH) cm^–1^; ^1^H NMR (400 MHz DMSO-*d*_6_, δ) 1.20–1.26 (m, 6H, CH_3_), 1.60–1.64 (m, 2H, CH_2_), 1.77–1.82 (m, 2H, CH_2_), 2.69 (t, *J* = 7.0 Hz, 2H, CH_2_), 4.09 (t, *J* = 7.0 Hz, 2H, CH_2_), 4.27 (q, *J* = 7.0 Hz, 2H, CH_2_), 6.93 (d, *J* = 8.0 Hz, 2H, benzene H), 7.19–7.24 (m, 7H, benzene H), 8.29 (s, 1H, alkene H), 10.65 (br s, 1H, NH). Anal. calcd for C_24_H_29_NO_5_: C, 70.05; H, 7.10; N, 3.40%. Found: C, 70.15; H, 7.11; N, 3.41%.

##### Diethyl 2-(((4-([1,1′-Biphenyl]-4-ylmethoxy)phenyl)amino)methylene)malonate (**10i**)

Compound **10i** was prepared from 2-(((4-hydroxyphenyl)amino)methylene)malonate by means of GP-A; 4-phenylbenzyl chloride; 2 h; *n*-hexane/ethyl acetate 70:30; ethanol; 69% as a white solid; 174 °C; IR ν 1710 (C=O), 2900 (NH) cm^–1^; ^1^H NMR (400 MHz DMSO-*d*_6_, δ) 1.13–1.17 (m, 6H, CH_3_), 4.03 (q, *J* = 7.0 Hz, 2H, CH_2_), 4.11 (q, *J* = 7.0 Hz, 2H, CH_2_), 5.07 (s, 2H, CH_2_), 6.98 (d, *J* = 8.0 Hz, 2H, benzene H), 7.23–7.30 (m, 3H, benzene H), 7.38 (t, *J* = 8.0 Hz, 1H, benzene H), 7.44–7.46 (m, 3H, benzene H), 7.58–7.61 (m, 4H, benzene H), 8.23 (d, *J* = 12.0 Hz, 1H, alkene H), 10.61 (d, *J* = 12.0 Hz, 1H, NH). Anal. calcd for C_27_H_27_NO_5_: C, 72.79; H, 6.11; N, 3.14%. Found: C, 72.65; H, 6.12; N, 3.13%.

##### Diethyl 2-(((4-((4-(Benzyloxy)benzyl)oxy)phenyl)amino)methylene)malonate (**10j**)

Compound **10j** was prepared from 2-(((4-hydroxyphenyl)amino)methylene)malonate by means of GP-A; 4-(benzyloxy)benzyl chloride; 3h; *n*-hexane/ethyl acetate 70:30; ethanol; 90% as a pink solid; 178 °C; IR ν 1718 (C=O), 2926 (NH) cm^–1^; ^1^H NMR (400 MHz DMSO-*d*_6_, δ) 1.12–1.18 (m, 6H, CH_3_), 4.01 (q, *J* = 7.0 Hz, 2H, CH_2_), 4.10 (q, *J* = 7.0 Hz, 2H, CH_2_), 4.92 (s, 2H, CH_2_), 5.03 (s, 2H, CH_2_), 6.92–6.95 (m, 4H, benzene H), 7.21–7.37 (m, 9H, benzene H), 8.23(d, *J* = 12.0 Hz, 1H, alkene H), 10.61 (d, *J* = 7.0 Hz, 1H, NH). Anal. calcd for C_21_H_23_NO_5_: C, 68.28; H, 6.28; N, 3.79%. Found: C, 68.34; H, 6.29; N, 3.80%.

#### Molecular Modeling

The X-ray crystal structure of the HIV-1 RNase H protein (PDB code 3QIP)^53^ was downloaded from the Protein Data Bank.^59,60^ The “Protein Preparation Wizard” tool of Maestro^61^ was employed to prepare the protein structure for the subsequent docking calculations. Specifically, hydrogens atoms were added according to their appropriate protonation states (pH = 7.4) and minimized, bond orders and disulfide bonds were calculated, and every water molecule was removed except for two conserved water molecules that participate in the magnesium ion chelation. The derivatives **4c**,**d**,**o**,**t** were virtually created through the Maestro 2D Sketcher tool. Then, with Ligprep, another utility present in the Schrödinger suite, also the ligands were prepared for the docking experiments, specifying that the ligands would participate in a chelation process. In particular, every hydrogen atom was added, and the appropriate tautomeric and ionization states were generated. Subsequently, the obtained ligands underwent a minimization with the OPLS3e force field.^62^ Then, the ligands were docked using Glide, part of the Schrödinger suite,^54^ which is a grid-based docking software that takes advantage of an energetic approach to find favorable interactions in the ligand–receptor complex. Multiple sets of fields are precalculated to render on a grid the structure of the receptor, along with its properties, to provide an accurate score of the ligand binding pose. For the grid generation, the grid box was placed at the mass center of the cocrystal ligand. This box provides a precise measure of the actual size of the search space. Nevertheless, ligands are allowed to leave the box boundaries in the course of grid minimization. The length of the outer box was fixed at 22 Å for the *X*, *Y*, and *Z* coordinates, while for the inner box, the default values of 10 Å were left. In the grid generation settings, it was specified that the ligand would preferentially chelate the magnesium ions. To dock compounds **4o** and **4t**, we employed Glide Induced Fit routine,^55^ which allows one to predict possible conformational changes in the binding site residues induced by the ligand binding process. Thus, during the docking process, an increased Coulomb–vdW cutoff and a reduced van der Waals radii are employed, along with a temporary removal of some of the most flexible side chains, to finally yield a diverse ensemble of ligand poses. For each of the generated poses, the residues’ side chains in the ligand proximity are reoriented to accommodate the ligand. Then, the ligand and the nearby residues undergo a minimization process. Finally, each ligand pose is redocked into its own minimized macromolecule, with each complex ranked according to its GlideScore. To dock compounds **4c** and **4d**, the standard Glide protocol with a rigid treatment of the protein was employed. On the whole, standard settings were used. The best-scoring complexes in terms of GlideScore were selected. All of the figures were rendered with the UCSF Chimera package.^63^

#### Magnesium Complexation Study

Complexation studies with MgCl_2_ were carried out on compounds **4o** and **5o**. The effects of the magnesium ion were evaluated by a spectrophotometric method, using a Perkin Elmer Lambda 40 UV–vis spectrophotometer and a Hellma quartz cuvette with a 1 cm optical path. Magnesium chloride (1 M solution) was purchased from Sigma-Aldrich (Milano, Italy) and was diluted with absolute ethanol to obtain stock solutions ranging from 4 10^–5^ to 0.2 M.

For the titration experiments, each studied compound was dissolved in 50–100 mL of absolute ethanol to the final concentration of 3.8 × 10^–5^ M for compound **4o** and 4.1 × 10^–5^ M for compound **5o**. Each solution (3 mL) was placed in a cuvette, and the UV–vis spectrum was recorded between 230 and 370 or 600 nm using ethanol as the reference. Thereafter, small volumes (10–15 μL) of the appropriate MgCl_2_ ethanolic stock solution were added both in the sample and in the reference cuvettes and the UV–vis spectra were recorded; the Mg^2+^ concentration in the solutions was increased from 0 to 100–200-fold with respect to the studied compound, in about 20 consecutive increments. Each experiment was conducted in triplicate.

To determine the stoichiometric coefficients of the complexes, Job’s method^64^ was used, which requires mixing, in appropriate proportions, equimolar solutions of metal ion and ligand, so that the final volume and the total moles present in the cuvette are equal for each measurement. The absorbance values were recorded at the wavelength where the higher difference in absorbance was observed in the UV–Vis titration experiments (320 nm for compound **4o** and 246 nm for compound **5o**). For each obtained absorbance value, the nominal absorbance values of equimolar solutions of the metal and ligand were subtracted, to obtain the Δ*A* due exclusively to the complex formation.

The resulting Δ*A* were reported in a graph as a function of the mole fraction of the ligand; the mole fraction *X*, which caused the maximum variation in absorbance, was determined by linear regression analysis and used to calculate the value of the coefficient *n*, which corresponds to the number of ligand molecules per cation, applying the following equation
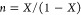


#### Expression and Purification of Recombinant HIV-1 RTs

His-tagged p66/p51 HIV-1 RT group M subtype B coded in a p6HRT-prot plasmid was expressed in the *Escherichia coli* strain M15.^65^ Heterodimeric RTs were expressed essentially and purified as described.^66^

#### Expression and Purification of Recombinant HIV-1 IN

His-tagged NL4-3 IN was expressed from a pET15b plasmid in the *E. coli* BL21 pLys strain. Recombinant protein was purified as previously described^67^ following a batch preparation on Ni-NTA beads (Qiagen, Paris, France).

#### Site-Directed Mutagenesis

Amino acid substitutions were introduced into the p66 HIV-1 RT subunit of a HIV-1 RT using a QuikChange mutagenesis kit, following the manufacturer’s instructions (Agilent Technologies Inc., Santa Clara, CA).

#### RNase H Polymerase-Independent Cleavage Assay

The HIV-1 RT-associated RNase H activity was measured as described.^68^ Briefly, the reaction was carried out in a black 96-well plate in a total volume of 100 μL. Serial dilutions of compounds were added to the reaction mix containing 50 mM Tris HCl pH 7.8, 6 mM MgCl_2_, 1 mM dithiothreitol (DTT), 80 mM KCl, 250 nM hybrid RNA/DNA (50-GTTTTCTTTTCCCCCCTGAC-30-Fluorescein, 50-CAAAAG AAAAGGGGGGACUG-30-Dabcyl). The reaction was started by the addition of 20 ng of HIV-1 wt RT, 20 ng of R448A RT, 20 ng of K451A RT, 40 ng of K540 RT, 60 ng of Q475A RT, 500 ng of N474A RT, 500 ng of Y501A RT, and 500 ng of W535A RT and incubated for 1 h at 37 °C. Products were quantified with a Perkin–Elmer Victor 3 multilabel counter plate reader at an excitation–emission wavelength of 490/528 nm. Experiments were performed in duplicate and replicated at least two times. Data were analyzed as described.^36^ Mean ± standard deviation of IC_50_ values were determined, and p values were calculated between the IC_50_ value against the wt and IC_50_ value against the mutants by paired, two-tailed *t* tests using GraphPad Prism 6.01 software (GraphPad Software, Inc.; San Diego, CA). Figures were made with GraphPad Prism 6 version 6.01.

### Polymerase Assay

The HIV-1 RT-associated RNA-dependent DNA polymerase activity was measured as described.^65^ Briefly, the reaction was carried out in a black 96-well plate in a total volume of 25 μL. Serial dilutions of compounds were added to the reaction mix containing 60 mM Tris HCl buffer pH 8.1, 8 mM MgCl_2_, 60 mM KCl, 13 mM DTT, 2.5 mM poly(A)-oligo(dT), and 100 mM dTTP. The reaction was started by the addition of 20 ng of HIV-1 wt RT and incubated for 30 min at 37 °C, followed by addition of 2 mL of 200 mM ethylenediamine tetraacetic acid (EDTA). Reaction products were detected by addition of 170 mL of revealing solution containing Picogreen in 10 mM Tris HCl pH 7.5, 1 mM EDTA, and measured with a multilabel counter plate reader Victor 3, equipped with filters 502/523 nm (excitation/emission wavelength).

### IN Assay

The DNA substrate was generated by annealing an equimolar amount of 19T (GTGTGGAAAATCTCTAGCA) and 21B (ACTGCTAGAGATTTTCCACAC). Both oligonucleotides were purchased from Integrated DNA Technologies, Inc. (Coralville, IA), and the gel was purified in-house. ST reactions were performed by adding molecules or an equivalent volume of 100% dimethyl sulfoxide (DMSO, used as the drug solvent) to a mixture of 20 nM duplex DNA substrate and 400 nM IN in 50 mM MOPS pH 7.2, 7.5 mM MgCl_2_, and 14 mM 2-mercaptoethanol. Mixtures were incubated at 37 °C for 2 h, and the reaction was quenched by addition of an equal volume of loading buffer [formamide containing 1% sodium dodecyl sulfate (SDS), 0.25% bromophenol blue, and xylene cyanol]. Reaction products were separated in 16% polyacrylamide denaturing sequencing gels. Dried gels were visualized using a FLA5000 (Fuji Photo Film, Tokyo, Japan). Densitometric analyses were performed using ImageQuant 5.1 software from GE Healthcare. Data analyses (linear regression, IC_50_ determination, and standard deviation) were performed using Prism 6.07 software from GraphPad (San Diego, CA).

### Cell-Based Assays

HIV-1 replication was monitored using HeLa-CD4-LTR-β-gal reporter cells as previously described.^69^ Briefly, β-galactosidase activity was monitored 24 h post infection using a replication-competent HIV produced in-house (H9-laï coculture) and a serial dilution of molecules or an equivalent volume of DMSO. In parallel, compounds’ toxicity was determined at 24 h in the same cell line using CellTiter 96 AQueous One Solution Cell Proliferation Assay (Promega, France).

## Supporting Information Available

Tables S1 and S2, Figures S1 and S2; details of HPLC analyses and HPLC traces of compounds **4o** and **5o** (PDF)
SMILES formulas for compounds **1–3**, **4a–t**, **5a–t**, **6**, **7**, **8a–c**,**e–t**, **9**, **10a–j**, **11**, and related data (CSV)
HIV RNase H in complex with docking poses of compound **4o** (PDB)
HIV RNase H in complex with docking poses of compound **4t** (PDB)

## Supplementary Material

jm1c00535_si_002.csv

jm1c00535_si_003.pdb

jm1c00535_si_004.pdb

## Abbreviations Used

AIDS: acquired immune deficiency syndrome
ART: antiretroviral therapy
DDDP: DNA-dependent DNA polymerase
DKA: diketo acid
EMME: diethyl ethoxymethylenemalonate
EWG: electron withdrawing
FDA: Food and Drug Administration
GP: general procedure
HIV-1: human immunodeficiency virus type 1
HPLC: high-performance liquid chromatography
IN: integrase
INSTI: integrase inhibitor
IR: infrared
NNRTI: non-nucleoside reverse transcriptase inhibitor
NRTI: nucleoside/nucleotide reverse transcriptase inhibitor
PI: protease inhibitor
RDDP: RNA-dependent DNA polymerase
RHI: RNase H inhibitor
RNase H: ribonuclease H
RT: reverse transcriptase
SAR: structure–activity relationship
UNAIDS: Joint United Nations Programme on HIV and AIDS
WHO: World Health Organization
