# Supplementary material for: Quinolinonyl Non-Diketo Acid Derivatives as Inhibitors of HIV-1 Ribonuclease H and Polymerase Functions of Reverse Transcriptase
Source: J Med Chem. 2021 Jun 9;64(12):8579–98. doi: 10.1021/acs.jmedchem.1c00535 (PMC8279492; doi:10.1021/acs.jmedchem.1c00535)
Supplement: Supplementary file 1 — jm1c00535_si_001.pdf [file jm1c00535_si_001.pdf]

SUPPORTING INFORMATION

Quinolinonyl Non-Diketo Acid Derivatives as Inhibitors of HIV-1 Ribonuclease H and Polymerase Functions of Reverse Transcriptase

Antonella Messore,[a^] Angela Corona,[b^] Valentina Noemi Madia,[a^] Francesco Saccoliti,[c] Valeria Tudino,[a] Alessandro De Leo,[a] Davide Ialongo,[a] Luigi Scipione,[a] Daniela De Vita,[d] Giorgio Amendola,[e] Ettore Novellino,[f] Sandro Cosconati,[e] Mathieu Métifiot,[g] Marie-Line Andreola,[g] Francesca Esposito,[b] Nicole Grandi,[b] Enzo Tramontano,[b] Roberta Costi,[a] and Roberto Di Santo,[a]*

[a] Dipartimento di Chimica e Tecnologie del Farmaco, Istituto Pasteur-Fondazione Cenci Bolognetti, "Sapienza" Università di Roma, p.le Aldo Moro 5, I-00185, Rome, Italy

[b] Department of Life and Environmental Sciences, University of Cagliari, Cittadella Universitaria di Monserrato, SS554 -09042 Monserrato (CA) Italy

[c] D3 PharmaChemistry, Italian Institute of Technology, Via Morego 30, I-16163 Genova, Italy

[d] Department of Environmental Biology, "Sapienza" University of Rome, p.le Aldo Moro 5, I-00185, Rome, Italy

[e] DiSTABiF, University of Campania "Luigi Vanvitelli", Via Vivaldi 43, 81100 Caserta, Italy

[f] Department of Pharmacy, University Federico II of Naples, Via D. Montesano 49, 80131 Naples, Italy

[g] Laboratoire MFP, UMR 5234, CNRS - Université de Bordeaux, 146 rue Léo Saignat, 33076, Bordeaux cedex, France

^These authors equally contributed.

*For R.C.: phone, +39-06-49693247; email, roberta.costi@uniroma1.it.

Table of contents

Table S1	S3
Table S2	S4
Figure S1	S5
Figure S2	S6
Details of HPLC analyses	S7
HPLC traces of compounds **4o** and **5o**	S8-S9
SMILES formulas for compounds **1-3, 4a-t, 5a-t, 6, 7, 8a-c,e-t, 9, 10a-j, 11** and related data	S10

Table S1. Effects of selected amino acid substitutions of HIV-1 RT in the susceptibility to compound **4o** in RNase H activity assays.

Cpd		wt	R448A	K451A	N474A	Q475A	Y501A	W535A	K540A
**4o**	IC_{50}[a]	5.2 ± 1.1	19.6± 1.24	11.8 ± 1.2	85.8 ± 1.6	>100	>100	>100	31.6 ± 1.2
	P value[b]		<0,0001	0,0002	<0,0001	<0,0001	<0,0001	<0,0001	<0,0001
			****	***	****	****	****	****	****
	Fold[c]		3.7	2.2	16.3	>19.1	>19.1	>19.1	6.0

The table reports the mean and standard deviation of three independent experiments. [a]Concentration (μM) required to inhibit HIV-1 RT-associated RNase H activity by 50% obtained by three independent experiments (reported as mean ± standard deviation). [b]p value calculated by a two-tailed T test using GraphPad Prism 6.01 software: p value < 0.05 (*); p value < 0.01 (**); p value < 0.001 (***); p value < 0.0001(****). [b]Fold increase in IC_{50} compared to wt RT.

Table S2. Effects of selected amino acid substitutions of HIV-1 RT in the susceptibility to compound **4t** in RNase H activity assays.

Cpd		wt	R448A	K451A	N474A	Q475A	Y501A	W535A	K540A
**4t**	IC_{50}[a]	1.9 ± 0.4	2.2 ± 1.3	2.9 ± 1.3	1.16 ± 1.41	15.6 ± 1.6	>100	>100	2.0 ± 1.2
	P value[b]		0,6354	0,1994	0,3438	< 0,0001	< 0,0001	< 0,0001	0,8268
			ns	ns	ns	****	****	****	ns
	Fold[c]		1.2	1.5	0.6	8.2	>52.4	>52.4	1.1

The table reports the mean and standard deviation of three independent experiments. [a]Concentration (μM) required to inhibit HIV-1 RT-associated RNase H activity by 50% obtained by three independent experiments (reported as mean ± standard deviation). [b]p value calculated by a two-tailed T test using GraphPad Prism 6.01 software: p value < 0.05 (*); p value < 0.01 (**); p value < 0.001 (***); p value < 0.0001(****). [b]Fold increase in IC_{50} compared to wt RT.

Figure S1. The HIV-1 RT-associated RNase H activity of point mutated HIV-1 RTs. The reaction was carried out in a black 96 well plate in a total volume of 100 µL. Serial dilutions of compounds were added to the reaction mix containing 50 mM Tris HCl pH 7.8, 6 mM $MgCl_2$, 1 mM dithiothreitol (DTT), 80 mM KCl, 250 nM hybrid RNA/DNA (50-GTTTTCTTTTCCCCCCTGAC-30-Fluorescein, 50-CAAAAG AAAAGGGGGGACUG-30-Dabcyl). The reaction was started by the addition of the corresponding dilutions of the enzymes and incubated for 1 h at 37 °C. Products were quantified with a Perkin–Elmer Victor 3 multilabel counter plate reader at excitation–emission wavelength of 490/528nm. Figures were made with GraphPad Prism 6 version 6.01.

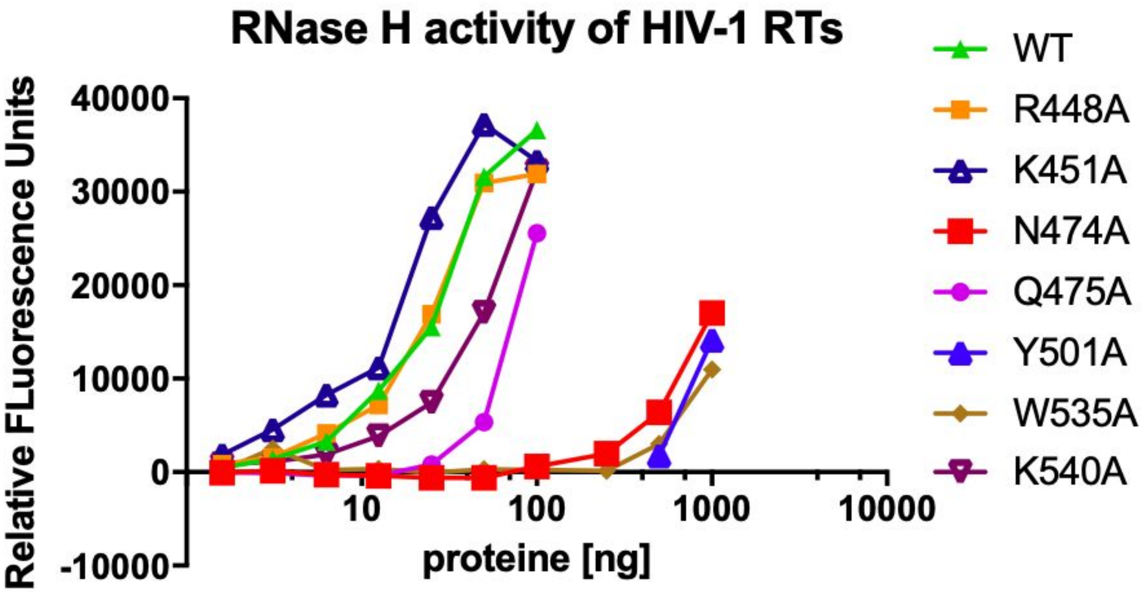

Figure S2. [A] UV spectra of **4o** in EtOH 4.23 10^{-5}M (black trace) and **4o** (4.23 10^{-5}M) + $MgCl_2$ (4.23 10^{-3}M) (red trace). [B] Increments of absorbance (A) at 256 nm obtained during the titration of **4o** with $MgCl_2$. [C] Job's plot obtained for **4o** and $MgCl_2$. ΔA at 320 nm was plotted *vs* molar ratio of **4o**, the maximum ΔA was observed at X = 0.52 which correspond a stoichiometry of 1:1 for the complex **4o** - Mg^{2+}. The Job's plots obtained at 240, 256 and 320 nm showed the maximum variation of absorbance at approximately 0.5 molar ratio of **4o**; 320 nm plot was used as it resulted less noisy and with a better alignment of the points.

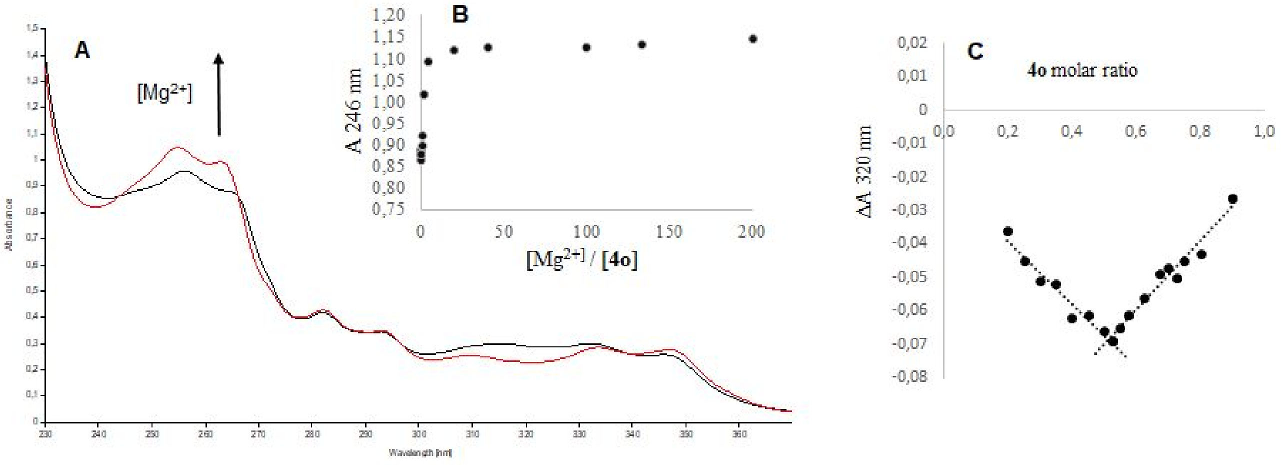

Details of HPLC analyses:

- Column: analytical column Waters Symmetry C18 (150 mm x 4.6 mm, 3.5 μm)
- UV detector: Full PDA; acquisition λ: 254 nm
- Eluent: isocratic flow acetonitrile: water 70:30 (0.1% TFA) *v/v* 1mL/min at 25°C.

HPLC trace of derivative **4o**.

==== Shimadzu LCsolution Analysis Report ====

C:\LabSolutions\Data\Project1\3042_1_CH3CN_07+H2O_03.lcd

Acquired by	: Admin
Sample Name	: 4042_1_CH3CN_07+H2O_03
Sample ID	: 4042_1_CH3CN_07+H2O_03
Vail #	: 0
Injection Volume	: 6 uL
Data File Name	: 3042_1_CH3CN_07+H2O_03.lcd
Method File Name	: chino_isocratica_CH3CN_07+H2O_03.lcm
Batch File Name	:
Report File Name	: Default.lcr
Data Acquired	: 27/04/2021 15.49.59
Data Processed	: 27/04/2021 16.05.01

<Chromatogram>

C:\LabSolutions\Data\Project1\3042_1_CH3CN_07+H2O_03.lcd

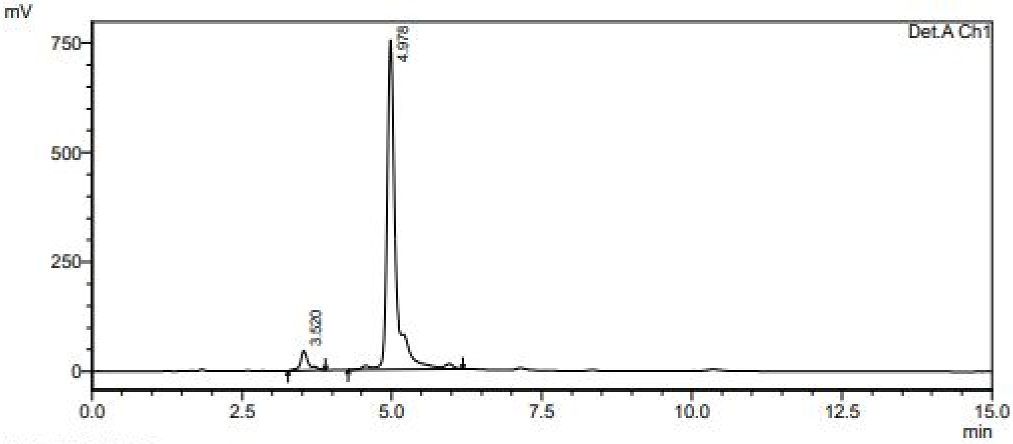

1 Det.A Ch1/254nm

PeakTable

Detector A Ch1 254nm

Peak#	Ret. Time	Area	Height	Area %	Height %
1	3.520	402757	44494	5.158	5.594
2	4.978	7405927	750906	94.842	94.406
Total		7808683	795400	100.000	100.000

HPLC trace of derivative **5o**.

==== Shimadzu LCsolution Analysis Report ====

C:\LabSolutions\Data\Project1\3041_2_CH3CN_07+H2O_03.lcd

Acquired by	: Admin
Sample Name	: 3041_2_CH3CN_07+H2O_03
Sample ID	: 3041_2_CH3CN_07+H2O_03
Vail #	: 0
Injection Volume	: 3 uL
Data File Name	: 3041_2_CH3CN_07+H2O_03.lcd
Method File Name	: chino_isocratica_CH3CN_07+H2O_03.lcm
Batch File Name	:
Report File Name	: Default.lcr
Data Acquired	: 28/04/2021 12.00.48
Data Processed	: 28/04/2021 12.15.51

<Chromatogram>

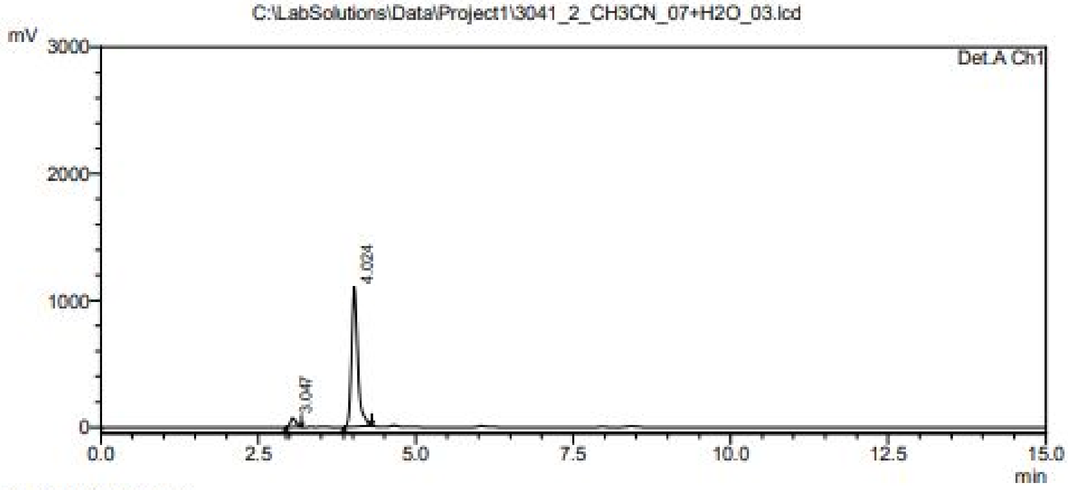

1 Det.A Ch1/254nm

PeakTable

Detector A Ch1 254nm

Peak#	Ret. Time	Area	Height	Area %	Height %
1	3.047	401230	70162	4.841	5.981
2	4.024	7886236	1103009	95.159	94.019
Total		8287466	1173171	100.000	100.000
